# Redox dysregulation as a driver for DNA damage and its relationship to neurodegenerative diseases

**DOI:** 10.1186/s40035-023-00350-4

**Published:** 2023-04-14

**Authors:** Sina Shadfar, Sonam Parakh, Md Shafi Jamali, Julie D. Atkin

**Affiliations:** 1 Centre for Motor Neuron Disease Research, Macquarie Medical School, Macquarie University, Sydney, NSW 2109 Australia; 2 La Trobe Institute for Molecular Science, La Trobe University, Bundoora, Melbourne, VIC 3086 Australia

**Keywords:** Redox dysregulation, DNA damage, Neurodegeneration, Reactive oxygen species, Oxidative stress

## Abstract

Redox homeostasis refers to the balance between the production of reactive oxygen species (ROS) as well as reactive nitrogen species (RNS), and their elimination by antioxidants. It is linked to all important cellular activities and oxidative stress is a result of imbalance between pro-oxidants and antioxidant species. Oxidative stress perturbs many cellular activities, including processes that maintain the integrity of DNA. Nucleic acids are highly reactive and therefore particularly susceptible to damage. The DNA damage response detects and repairs these DNA lesions. Efficient DNA repair processes are therefore essential for maintaining cellular viability, but they decline considerably during aging. DNA damage and deficiencies in DNA repair are increasingly described in age-related neurodegenerative diseases, such as Alzheimer’s disease, Parkinson’s disease, amyotrophic lateral sclerosis and Huntington’s disease. Furthermore, oxidative stress has long been associated with these conditions. Moreover, both redox dysregulation and DNA damage increase significantly during aging, which is the biggest risk factor for neurodegenerative diseases. However, the links between redox dysfunction and DNA damage, and their joint contributions to pathophysiology in these conditions, are only just emerging. This review will discuss these associations and address the increasing evidence for redox dysregulation as an important and major source of DNA damage in neurodegenerative disorders. Understanding these connections may facilitate a better understanding of disease mechanisms, and ultimately lead to the design of better therapeutic strategies based on preventing both redox dysregulation and DNA damage.

## Background

All types of cells in the human body require oxygen for their physiological functions. However, the brain displays particularly high rates of metabolic activity, and it consumes up to 20% of available oxygen, much more than other organs [1]. Oxygen is highly reactive with other molecules and oxidation refers to the transfer of electrons from an atom to oxygen, with the formation of a negative ion. Reduction is the opposite process, referring to a gain of electrons. The delicate balance between cellular oxidation and reduction reactions, referred to as the cellular ‘redox state’, must always be maintained. However, imbalance in the redox state leads to the formation of free radicals, including reactive oxygen species (ROS), reactive nitrogen species (RNS) and reactive sulphur species (RSS) [2, 3]. Low levels of ROS, RNS and RSS are necessary for proper functioning of fundamental cellular processes such as proliferation, host defence, signal transduction, and gene expression (4, 5). However, excessive amounts of ROS, RNS and RSS can be severely toxic to cells. To neutralize the destructive effects of these species, the cell employs antioxidant systems to minimize oxidative damage. The cellular redox state therefore represents an essential defence system that regulates numerous signalling pathways, including DNA repair, calcium metabolism, axonal transport and protein homeostasis (proteostasis) mechanisms such as protein folding and degradation [6]. However, dysregulation of redox conditions disrupts these processes and can lead to aberrant post-translational modification of redox-sensitive proteins [6].

Dysregulation of cellular redox conditions is a major source of DNA damage because redox homeostasis activates or inhibits key proteins involved in DNA repair. Eukaryotic cells have developed complex signalling mechanisms, together referred to as the ‘DNA damage response’ (DDR), to detect, signal and repair DNA damage and thus maintain genome integrity [7]. However, if DNA lesions remain unrepaired, the accumulating DNA damage induces various cell death mechanisms to eradicate those cells with imperfect genomes. Whilst the DDR itself has now been characterised in some detail, the relationship between DNA damage and the cellular redox state is poorly understood in comparison and has emerged relatively recently.

Neurodegenerative diseases are devastating conditions that result from chronic degeneration and death of specific types of neurons. Whilst most cell types are continuously replaced and thus can withstand the loss of cells displaying irreparable DNA damage by apoptosis, neurons are post-mitotic and therefore susceptible to DNA lesions throughout their lifespan. Hence, they are particularly susceptible to damage. In addition, compared to other cell types, neurons are remarkably vulnerable to redox dysregulation due to their excessive oxygen consumption, large size, and high rates of metabolism, which produces significant quantities of ROS and RNS [8]. Age-associated increases in redox dysfunction contribute to protein misfolding and aggregation, and are widely implicated in neurodegeneration [9]. Furthermore, aging is the most significant risk for neurodegenerative diseases, and redox homeostasis and the efficiency of DNA repair become significantly impaired during aging. Not surprisingly, dysregulation of the cellular redox state has been widely described in neurodegenerative conditions, including Alzheimer’s disease (AD) [10], Parkinson’s disease (PD) [11], amyotrophic lateral sclerosis (ALS) [12–14] and related condition frontotemporal dementia (FTD) [15], and Huntington’s disease (HD) [16]. Furthermore, impaired repair of DNA damage is now strongly linked to age-associated neurodegenerative diseases [17–19]. Moreover, there have been major advances in this field over the last five years. Hence in this review, we provide a comprehensive and updated appraisal of current knowledge relating redox dysfunction to DNA damage, and discuss how this is impacted in neurodegenerative disorders.

## DNA damage

Preservation of genetic material is essential for the perpetuation of life [7], but DNA is continuously subject to both exogenous and endogenous threats [7, 20]. In fact, it has been estimated that every day most human cells are exposed to tens of thousands of DNA lesions [21, 22]. Unrepaired DNA damage leads to mutations, compromises cellular viability, and prevents the correct transfer of genetic material to the next generation [22]. Many cellular functions, including DNA replication and transcription, are dysregulated following failure to repair DNA [7, 20]. Conversely, genome abnormalities, mutations and cell death can result from hindered DNA replication or transcription [7, 23]. To protect the genome, cells use the DDR to prevent or tolerate distinct types of DNA damage [20, 21, 24].

The mammalian DDR involves several components: (a) mechanisms to repair DNA to minimise the damage and thus restore the fidelity of genetic material; (b) activation of DNA damage checkpoints to arrest the cell cycle, thus providing more time for DNA repair to prevent the transfer of damaged DNA to daughter cells; (c) induction of a transcriptional response to allow expression of specific genes; and (d) apoptosis, to eliminate critically damaged cells, and therefore protect the organism [20]. Below, we discuss how redox-regulated mechanisms control the functions of the DDR. For a more detailed discussion of specific DDR mechanisms, please see several excellent recent review articles on this topic [7, 25–27].

## The cellular redox system

**Fig. 1:**
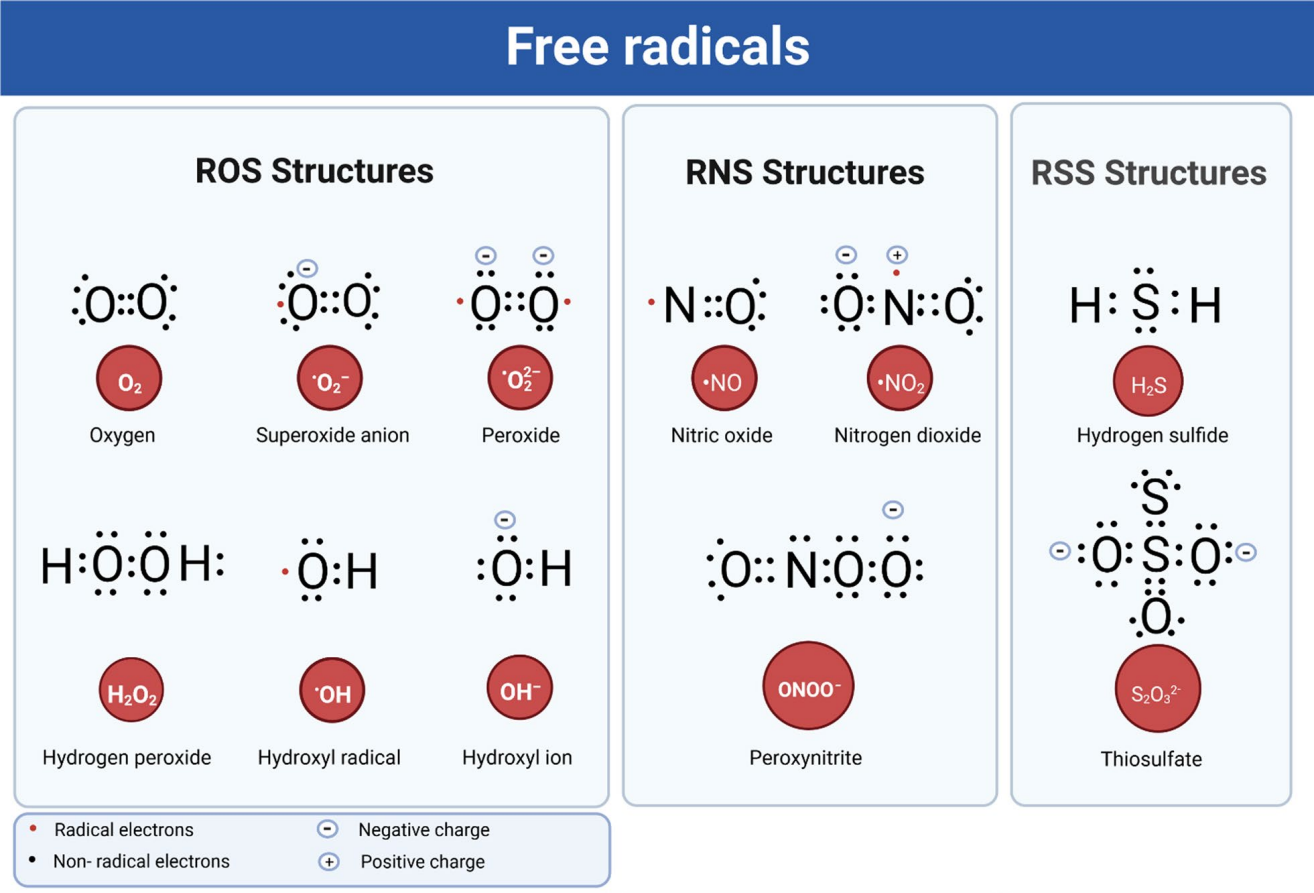
Lewis structures of free radicals. Free radicals are highly reactive molecules with an uneven number of electrons that have the potential to harm cells. ROS, including ·OH, O_2_^·−^, ^1^O_2_, ·HO_2_ and H_2_O_2_, are types of free radicals containing oxygen. RNS are highly active molecules derived from nitric oxide-derived compounds including ·NO, ONOO^−^, and ·NO_2_. RSS are a family of sulphur-based chemical compounds that include H_2_S and S_2_O_3_^2−^ that can oxidize and inhibit thiol-proteins and enzymes

The cellular redox system involves the production of free radicals—highly reactive molecules containing an uneven number of electrons [28]—and the antioxidant processes that neutralize them. An imbalance of these reactive species leads to oxidative or nitrosative stress. ROS include hydroxyl radicals (·OH), superoxide (O_2_^·−^), singlet oxygen (^1^O_2_), hydroperoxyl radicals (·HO_2_) [28, 29] and hydrogen peroxide (H_2_O_2_) [30]. In addition, peroxyl radicals (ROO·) are carbon-centred free radicals that are also classified as ROS [31]. O_2_^·−^ is the origin of most intracellular ROS, but it is transformed either to H_2_O_2_ by the activity of catalase, or to peroxynitrite (PN) (ONOO^−^) by reaction with nitric oxide ·NO [30]. RNS include NO-derived compounds, including nitric oxide (·NO), PN (ONOO^−^), and nitrogen dioxide (·NO_2_) [30]. RSS are commonly produced by the oxidation of thiols and disulphide into higher oxidation states and they include persulphate, polysulphide, and thiosulphate (S_2_O_3_^2−^) [32] (Fig. 1). Free radicals can attack different cellular components in neurons, including DNA, proteins and lipids, rendering them susceptible to oxidative stress. The highly reactive ·OH radical in particular damages both heterocyclic DNA bases and the sugar moiety [33].

**Fig. 2:**
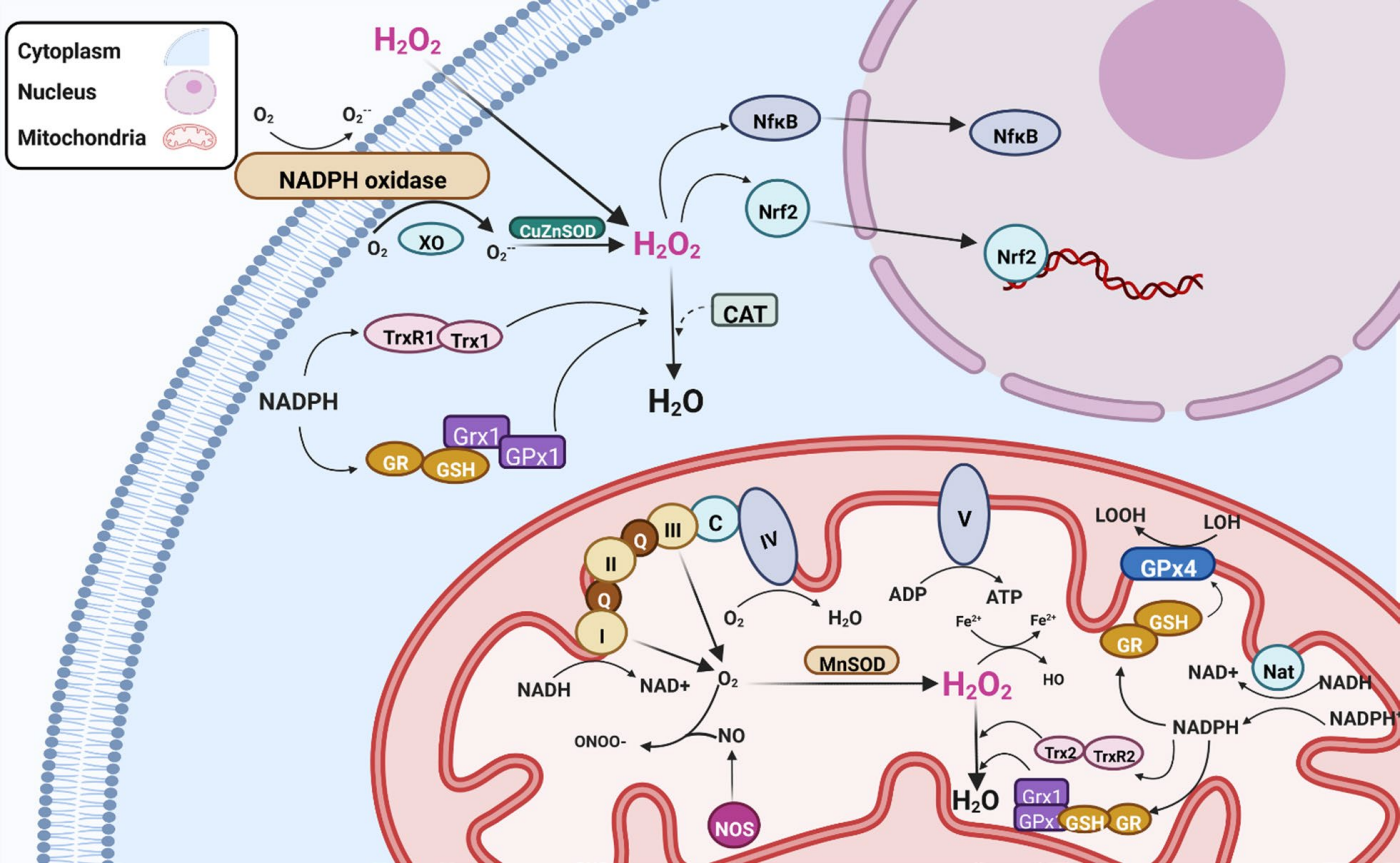
Mechanisms involved in maintaining cellular redox homeostasis. The cellular redox state is a sensitive balance between oxidation and reduction reactions, involving the production of free radicals and the antioxidant systems that neutralize them. H_2_O_2_ is generated by SOD enzymes CuZnSOD in the cytoplasm, and it also enters the cell from the extracellular space, which together enhance intracellular H_2_O_2_ levels. H_2_O_2_ can be safely decomposed by catalase into water (H_2_O) and oxygen (O_2_). The mitochondrial enzyme MnSOD also has dismutase activity, which detoxifies the free radical O_2_^·−^ generated by mitochondrial respiration. The cellular redox state is regulated by the thioredoxin (Trx/TrxR) and glutaredoxins (Grx) systems, which modifies specific redox-sensitive proteins, thereby triggering related signalling events. The nuclear factor erythroid 2-related factor 2 (Nrf2) system is then activated, leading to an antioxidant response. The NADPH oxidase complex is inactive under normal circumstances but is activated during respiratory burst. Glutaredoxin 4 (GPx4) reduces lipid hydroxide (LOOH) to alcohol (LOH). *GR* glutathione reductase, *NF-κB* nuclear factor kappa B, *NOS* nitric oxide synthase, *NADPH* nicotinamide adenine dinucleotide phosphate

Cells have established complex antioxidant systems to defend against oxidative insults, involving both enzymes and cofactors that maintain redox balance, and mechanisms to limit respiration in mitochondria. Endogenous antioxidant enzymes include superoxide dismutases (SOD), catalase, glutathione peroxidase (GPx), glutathione reductase (GR), and peroxiredoxins (Prxs) [34, 35]. Smaller antioxidant molecules include glutathione, coenzyme Q, ferritin, bilirubin, ascorbic acid (vitamin C), and α-tocopherol (vitamin E) [36]. The overall cellular redox state is determined by two cellular disulphide mechanisms, the thioredoxin (Trx) and glutaredoxins (Grx) systems [37]. Free radicals affect many cellular components (proteins, nucleic acids, lipids, and carbohydrates). However, in this review we will focus only on those molecules relevant to DNA damage (Fig. 2).

### Cellular redox mechanisms

Both Grx and Trx enzymes belong to the Trx superfamily, whose members are characterised by the presence of an active-site Cys-X-X-Cys motif in a Trx-like fold [38]. These antioxidant enzymes regulate the activity of substrate proteins through alterations of the redox state of thiol groups within their active-site cysteines. These thiols can be either reduced, or oxidized, where the two cysteines form an intramolecular disulphide bond with substrate proteins. Grxs and Trxs are present in multiple organelles, including the nucleus, and they often shuttle between the nucleus and cytoplasm [39].

Grxs are small enzymes that use glutathione (GSH) as a co-factor to maintain their reduced state [40]. GSH is a tripeptide consisting of γ-l-glutamyl-l-cysteinyl-glycine, which is present at high concentrations in most cells, including neurons [41]. GSH exists in both reduced and oxidized (GSSG) states and GSH regulates the thiol-disulphide redox states of proteins by maintaining their sulfhydryl groups in a reduced form. Hence the ratio of GSH to GSSG determines the cellular redox status. In normal cells the GSH/GSSG ratio is > 100, whereas in conditions of oxidative stress, this ratio decreases to > 10. GSH also participates in many antioxidant defence reactions including the synthesis of nucleic acids [42]. A unique feature of Grxs is their ability to catalyse the addition of GSH to a substrate protein (glutathionylation), and the reverse reaction (deglutathionylation), which together can also regulate redox conditions. The Trx system also consists of nicotinamide adenine dinucleotide phosphate (NADPH), GR, GPx, and Grx [37]. NADPH is the fundamental reductant that maintains the redox states of both the Trx and Grx systems. DNA repair is dependent on GSH, since elevation of DNA damage is related to defects in GSH metabolism in mice [43].

The GSH pool of the nucleus is an important protective factor against DNA damage induced by oxidation [44]. It also protects nuclear proteins in this reducing environment, facilitating gene transcription throughout the cell cycle in dividing cells [45]. Elevated GSH levels result in deglutathionylation of DNA-repair proteins, and hence more repair and protection against DNA damage [46]. GSH and Grx are protective against oxidative DNA damage through the regulation of DNA repair enzymes [47, 48]. GSH is synthesized in neurons and protects DNA from oxidative stress in the brain [49]. However, the molecular functions of GSH in the neuronal nucleus and how GSH is transported to the nucleus in neurons, remain topics of debate.

The main function of Trx is the reduction of cysteines and cleavage of disulphide bonds in substrate proteins. In addition to Trx and NADPH, the Trx system also comprises thioredoxin reductase-1 (Txnrd1), which maintains Trx proteins in their reduced state via NADPH [12]. Similar to the Grx system, Trx has been implicated in DNA repair [50, 51]. Thioredoxin-1 (Trx1) is protective against oxidative DNA damage through the regulation of DNA repair enzymes [47]. In addition, Trx1 plays a major role in the reduction of apurinic/apyrimidinic endonuclease 1 (APE1) [52]. Impaired DNA repair and cell cycle arrest have been reported following impaired activity of 2′-deoxyribonucleotides in Txnrd1-deficient T cells, implying that Txnrd also functions in the DDR [53]. Overexpression of thioredoxin-interacting protein (TXNIP), a negative regulator of Trx [51], elevates oxidative DNA damage and shortens lifespan in *Drosphilia*, while downregulation of TXNIP increases resistance to oxidative stress and extends lifespan [51]. Under physiological conditions, cytosolic Trx1 interacts with apoptosis inducing factor (AIF), although this is disrupted following the induction of oxidative stress [54]. Furthermore, the interaction between AIF and DNA is impaired following localization of Trx1 in the nucleus, thus attenuating AIF-mediated DNA damage [54].

Protein disulphide isomerase (PDIA1, also known as PDI) is the prototype member of a large family of Trx proteins that possess two different activities: disulphide interchange/oxidoreductase function, involving oxidation, reduction and/or isomerisation of protein disulphide bonds, and general chaperone activity [55]. Hence, PDI catalyses the correct folding of misfolded or unfolded proteins into their native structure. PDI and Erp57, the family member with closest homologue to PDI, facilitate disulphide bond formation in almost all cellular proteins [56]. PDI is upregulated during the unfolded protein response (UPR), where it alleviates endoplasmic reticulum (ER) stress by enhancing protein folding [57]. However, whilst PDI is conventionally regarded as being localised in the ER, it has been detected in other cellular locations, including the nucleus [58, 59].

In addition to these specific enzyme systems, mitochondria are the major organelles that regulate redox reactions. They are the main site of energy production in cells via oxidative phosphorylation (OXPHOS) [60]. In fact, being the powerhouse of the cell, mitochondria provide approximately 80% of energy requirements, although they consume 90% of cellular oxygen [1]. Five distinct multiprotein complexes (I–V) comprise the mitochondrial OXPHOS system [61], and O_2_^·−^ is generated primarily by complexes I and III [62].

## Oxidative and nitrosative stress

Several organelles and cellular processes, as well as environmental agents, contribute to the generation of ROS. Under physiological conditions, ROS are beneficial because they are essential for many biological functions that depend on redox signalling. However, when redox conditions are dysregulated, they can be harmful. Mitochondria are the major source of oxidative stress, which can be detrimental by damaging both mitochondrial DNA (mtDNA) and proteins [63]. The mitochondrial genome is highly vulnerable to damage because unlike nuclear DNA, it is not protected by histones, and it is physically located close to the electron transport chain (ETC) [64, 65]. Hence, equivalent levels of free radicals can induce more lesions in mtDNA compared to nuclear DNA [66]. In addition, damage to mtDNA hinders expression of proteins involved in the ETC, dysregulating their activity, producing free radicals and disrupting mitochondrial functions [67].

Other physiological processes and proteins can produce ROS, such as xanthine oxidoreductase (XOR). Mammalian XOR catalyzes the conversion of hypoxanthine to xanthine, and further to uric acid during purine metabolism, generating H_2_O_2_ [68]. XOR activity can therefore induce the generation of ROS, resulting in oxidative DNA damage and cell death [69]. Other mechanisms such as peroxisomal metabolism, anabolic processes and catabolic oxidation, can also produce ROS as by-products [70]. Neurons rely heavily on accurate DNA repair mechanisms and efficient DDR due to their high metabolic rate, but this can also generate ROS and hence oxidative DNA damage [71].

Similarly, RNS can be either destructive or favourable to cells depending on the conditions. Whilst they regulate important physiological processes, RNS also can be toxic by damaging metabolic enzymes and by reaction with superoxide, generating PN [72]. Furthermore, the interaction between NO– and O_2_^·−^ creates the much more potent oxidant ONOO^−^, which influences whether NO induces physiological or pathological conditions [73]. PN binds to lipids, DNA, and proteins directly via oxidative reactions, or indirectly via radical-mediated mechanisms [73], and this can induce DNA damage [74].

ER stress can also induce redox dysfunction in cells [75, 76], which is increasingly linked to DNA damage [77, 78]. ER stress arises after accumulation of misfolded proteins in the ER, inducing the UPR [75]. Whilst these processes will not be discussed here, the reader is directed to several excellent recent review articles on this topic [75–79].

## Types of DNA damage

DNA is a highly reactive molecule [20, 80] and therefore very susceptible to injury. DNA can be damaged in several different ways. This involves modification or loss of individual bases, breakage of one or both DNA strands, or DNA replication errors, including topoisomerase-mediated damage. Single-stranded breaks result in gaps in a single strand of the DNA double helix, and they arise frequently (tens of thousands per cell per day). They are generally accompanied by loss of a single nucleotide and by damaged 5′- and/or 3′-termini at the site of the break [81]. In contrast, damage to both strands of DNA results in a double-stranded break (DSB) which is considered to be the most toxic DNA injury, because it can lead to cell death if unrepaired, and to chromosomal translocations if mis-repaired [82].

**Fig. 3:**
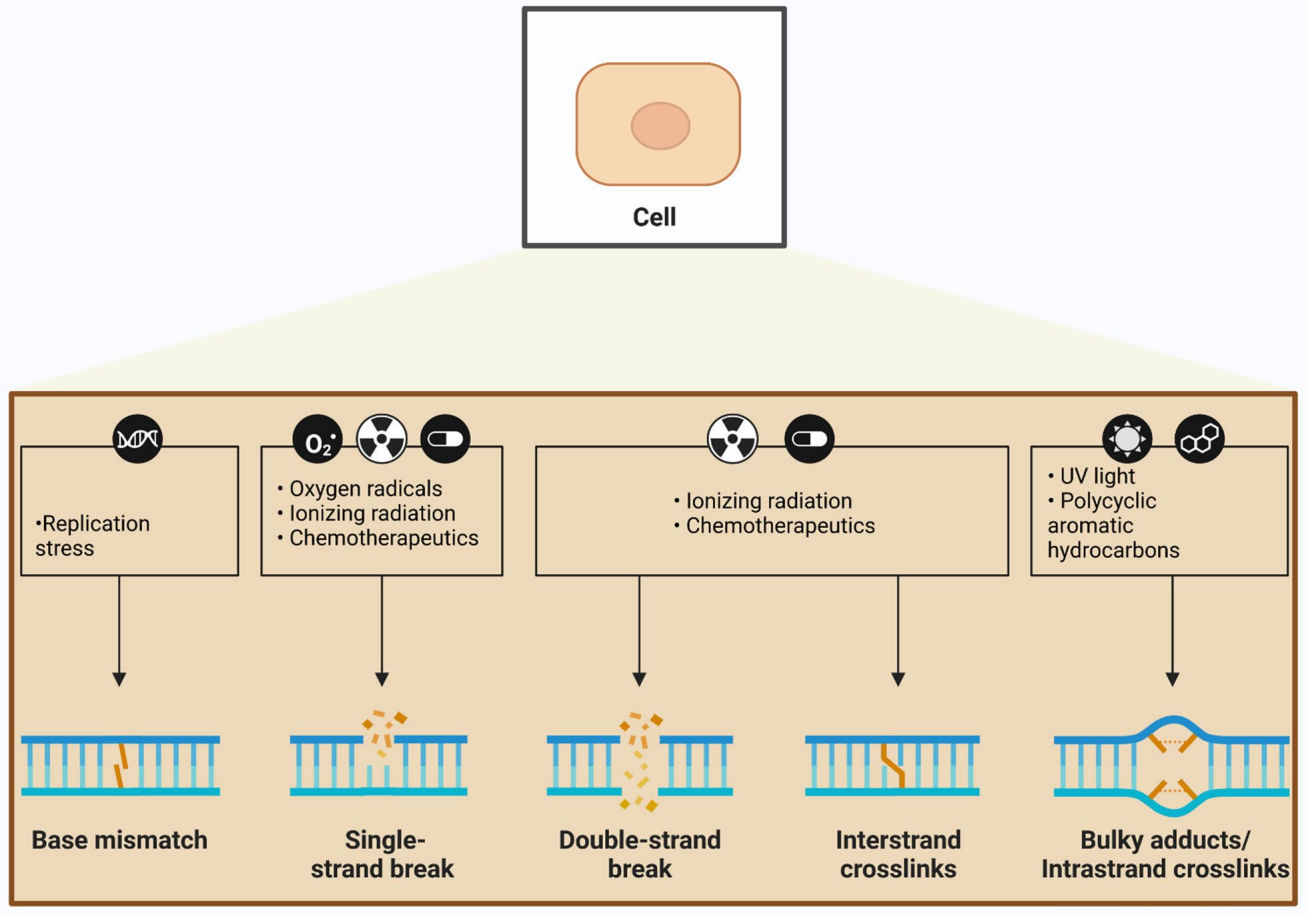
Types of oxidative DNA damage. Several types of stressors can lead to oxidative DNA damage. Replication stress is the major source of base mismatches in DNA, whereas free radicals primarily induce single-strand breaks (SSBs), and double-strand breaks (DSBs) to a lesser extent. Ionizing radiation and chemotherapeutics can induce both SSBs and DSBs, as well as interstrand crosslinks. DNA damage induced by UV radiation results in bulky DNA adducts

Insults to DNA can be categorized as either endogenous or exogenous (environmental) depending on the source of damage, but a major source of both endogenous and exogenous DNA damage is oxidative stress [83]. The most common forms of DNA damage resulting from redox dysregulation include SSBs, oxidative modification of bases, and the formation of apurinic/apyrimidinic (AP) or abasic sites, which are regions of DNA lacking either a purine or a pyrimidine base. Oxidative DNA damage can also involve base mismatches, DSBs, and inter-strand crosslinks (ICLs) (Fig. 3). However, as ROS mainly induce SSBs, DSBs may be the result of conversion of SSBs and/or result of oxidized bases or abasic sites during the DNA repair process [84]. Elevated ROS and RNS can also induce DNA-DNA or DNA–protein cross-linking and sister chromatid exchange, and translocation in nuclear DNA [85, 86] (Fig. 3). Replication stress, oxygen radicals, ionizing radiation (IR), chemotherapeutics, ultraviolet (UV) light, and polycyclic aromatic hydrocarbons (PAHs), can also initiate oxidative DNA damage. Oxidized bases are resolved primarily by base excision repair (BER), whereas the DNA backbone is repaired by SSB repair or DSB repair pathways [82, 87]. Below we discuss the possible sources of redox-relevant DNA damage and the types of damage that can result, as well as the mechanisms that repair these forms of damage.

### Exogenous DNA damage

Environmental conditions, including hypoxia, extreme temperatures (heat or cold) and oxidative stress, are important sources of exogenous DNA damage [88, 89]. Furthermore, many factors present in the environment can induce oxidative DNA damage. These include various types of radiation, chemical mutagens from food and other sources, industrial chemicals, and smoke.

#### UV radiation

UV radiation is one of the most powerful and carcinogenic environmental agents that interacts with DNA and can modify genomic integrity, either directly or indirectly [90]. UV radiation initiates the ‘preparation for oxidative stress’ antioxidant response, whereby antioxidants are upregulated [91] and the resulting minor redox imbalance leads to increased tolerance to additional oxidative insults. UV radiation also produces free radicals that attack the intracellular domains of ret tyrosine kinase, which is implicated in oncogenesis, leading to its dimerization and activation [92].

Usually UV radiation is divided into three categories based on the emission wavelength: UV-A (320–400 nm), UV-B (290–320 nm) and UV-C (190–290 nm) [90]. Both UVA and UVB radiation (to a lesser extent) induce oxidative DNA damage, unlike UV-C. UV radiation also induces DNA strand breaks and DNA–protein crosslinks [93], and UV-A radiation can target bases by photodynamic effects which involve the participation of singlet oxygen (^1^O_2_), and to a lesser extent, ^·^OH. To repair UV radiation-based damage, cells employ several defence mechanisms, including nucleotide excision repair (NER), homologous recombination, direct reversal of UV-damaged bases, and ICL repair [94, 95].

#### IR

IR is another source of exogenous DNA damage, which includes micro- and radio-waves, and alpha-, beta-, gamma-, and X-rays [96, 97]. IR is produced from the surroundings, including soil, rock, radon, medical devices and cosmic radiation [96]. Based on the quantity of energy transferred, IR radiation can be classified into either high linear energy transfer (LET), which refers to alpha radiation, or low LET, in the case of beta and gamma radiation [97].

Like UV radiation, DNA damage induced by IR can also be either direct or indirect (although it is mostly indirect) and associated with oxidative stress. Whilst IR directly induces DNA breaks, especially DSBs, it can also produce oxidative lesions in DNA by ROS, including the generation of abasic sites and SSBs [98], and by stimulating inducible nitric oxide synthase activity, thereby generating large amounts of ^·^NO. NO reacts with O_2_, producing ONOO^−^, which is highly invasive and induces DNA damage. Interestingly, SSBs formed by IR contain 3’ phosphate or 3’-phosphoglycolate ends instead of 3’ OH ends, and this differentiates them from other non-IR-induced SSBs [99]. DSBs can also be produced following adjacent sites damaged by IR that are present on both DNA strands [82]. IR-induced lesions are repaired by homologous recombination for DSBs [99], or by AP endonucleases, polynucleotide kinase phosphatase (PNKP) and tyrosyl DNA phosphodiesterase 1 (TDP1) for SSBs [100].

#### Chemical mutagens

In the sections below we discuss the mutagens known to induce oxidative DNA damage.

##### Alkylating agents

Alkylating agents are reagents that add alkyl groups to DNA bases, most commonly to guanine. Dietary ingredients, tobacco smoke, chemotherapeutic agents, burning biomass and industrial manufacturing are the foremost sources of exogenous alkylating agents [101], but this also includes sulphur and nitrogen mustards used in war [20]. Alkylation results in the formation of DNA adducts [20], including methyl methanesulfonate, ethyl methanesulfonate, methylnitrosourea and N-methyl-N’-nitro-N-nitrosoguanidine [102]. Nitrogenous base rings and the N3 of adenine and N7 of guanine are particularly susceptible to electrophilic alkylating agents, although all DNA bases are vulnerable [103]. The pathways involved in DNA repair induced by alkylated bases include BER, ICL and direct damage reversal pathways [102].

##### Aromatic amines

Aromatic amines are organic compounds containing an aromatic ring attached to an amine group that can induce oxidative DNA damage. They are present primarily in tobacco smoke, pesticides, motor fuels, and colourants. The most widely studied aromatic amines in vitro are 2-aminofluorene (AF) and N-acetyl-2-aminofluorene (AAF; an acetylated derivative of AF) [104]. Aromatic amines can be converted into esters and sulphates, modifying the C8 position of guanine, via alkylation by the activated P450 mono-oxygenase system, the primary cellular mechanism responsible for clearance of pharmacological compounds [105]. Oxidative DNA damage resulting from aromatic amines [106] is repaired by NER [107, 108].

##### PAHs

PAHs are non-polar hydrocarbons with two or more aromatic rings that are sources of DNA damage [20]. They include anthracene, naphthalene, pyrene, dibenzo [a,l] pyrene and benzo(a)pyrene [109]. The main sources of PAH in the environment are tobacco smoke, automobile exhaust fumes, incomplete combustion of organic materials, fossil fuels and overcooked food [110]. Similar to aromatic amines, exposure to PAHs promotes oxidative damage [111, 112], oxidative stress and lipid peroxidation [113]. The NER and BER pathways are involved in repair of PAH-induced DNA damage.

### Endogenous DNA damage

DNA damage can also arise spontaneously from natural metabolic processes, and most endogenous DNA lesions are SSBs (75%). Oxidative lesions form a major component of this form of DNA damage [114, 115]. We discuss below the major types of endogenous DNA damage related to cellular redox processes.

#### Base modifications

ROS, particularly the OH· radical, directly attack both purine and pyrimidine bases and the deoxyribose sugar backbone of DNA [28]. The OH· radical removes hydrogen atoms and generates modified purine and pyrimidine base by-products and DNA–protein cross-links [28]. Approximately 20 different oxidized base adducts can be generated by oxidative DNA damage induced by ROS [116]. Pyrimidine bases modified by OH· can produce distinct adducts such as uracil glycol, 5-hydroxydeoxy uridine, thymine glycol 5-hydroxy deoxycytidine, 5-formyl uracil, cytosine glycol, 5,6-dihydrothyronine, 5-hydroxy-6-hydro-cytosine, 5-hydroxy-6-hydro uracil, uracil glycol, alloxan and hydantoin [116]. Other adducts—8-hydroxydeoxy guanosine, 8-hydroxy deoxy adenosine, 2,6-diamino-4-hydroxy-5-formamidopyrimidine—can also be attacked by the purine adducts formed by OH·. Guanine is a prime target of oxidative DNA damage due to its lower reduction potential compared to other bases [83]. OH· radicals interact with the C4, C5 and C8 positions of the imidazole ring of guanine (G) to form several potentially mutagenic DNA lesions, including 8-oxoguanine (8-oxoG) [83]. This modified base pairs with adenine (A) instead of cytosine (C), leading to the frequent incorporation of mutations. PN can also interact with G to form 8-nitroguanine, which is used as a nitrosative DNA damage marker [83]. The deoxyribose sugar backbone of DNA can also form a number of free radical-induced adducts, including glycolic acid, 2-deoxytetrodialdose, erythrose, 2-deoxypentonic acid lactone, and 2-deoxypentose-4-ulose [116].

#### Base deamination

The deamination, or removal of an amino group from a base, is a major source of spontaneous DNA damage. In human cells, A, G, C, and 5-methyl cytosine (5mC) are capable of being deaminated, which convert to hypoxanthine, xanthine, uracil (U), and thymine (T), respectively [117]. Among these, 5mC becomes deaminated most frequently, followed by C [117], and single-stranded DNA (ssDNA) is the preferred target compared to double-stranded DNA (dsDNA) [118, 119]. Base deamination eventually induces mutations after successive DNA replication cycles. Oxidative stress is a major trigger of deamination and thus DNA damage [120–122]. However, deamination can also result from exposure to UV radiation, nitrate, sodium bisulphite and intercalating agents. DNA bases damaged by deamination are predominantly repaired by the BER pathway [123].

#### Abasic (apurinic/apyrimidinic) sites

The formation of an apurinic/apyrimidinic (AP), or abasic, site is one of the most frequent endogenous DNA lesions, particularly following oxidative stress [124]. Abasic sites result from the spontaneous hydrolysis or cleavage of N-glycosyl bonds, which link nitrogen bases to the sugar-phosphate backbone [20]. Human cells generate abasic sites at a higher frequency (approximately 1000 per day) compared to other organisms, which is further increased by high temperatures and extremes of pH, both acidic and basic [125]. Whilst oxidative stress promotes the formation of abasic sites, they are usually unstable and instantly convert into SSBs [126]. Abasic sites are principally repaired by BER, and sometimes by NER.

#### Topoisomerase (TOP)-mediated DNA damage

DNA TOP enzymes catalyze alterations of the topological state of DNA, and they are required for several important cellular functions, including DNA replication and transcription. The interweaved, supercoiled nature of the DNA double-helix can lead to topological problems and tension, but the introduction of transient breaks by TOPs allows the DNA strands to be rotated, thus relieving topological stress [127, 128]. However, this process can lead to endogenous DNA damage when these transient breaks are not repaired. Human TOPs are targets for some of the major chemotherapy drugs that function by inducing redox stress, producing ROS and lipid peroxidation products [129, 130]. TOP1 contains eight cysteines, two of which play a critical role in catalytic activity and are the target of thiol-reactive compounds [131].

There are two types of TOP enzymes: type I and type II, which act on SSBs and DSBs, respectively. In the case of topoisomerase type 1 (TOP1), temporary nicks wrap around TOP1-bound DNA, forming a complex that relaxes the DNA. TOP1 aligns the 5’-OH group of the DNA with the tyrosine-DNA phosphodiester bond to ligate the nicked ends and thus resolve the complex [132, 133]. Hence, stabilization of DNA breaks induces DNA damage, resulting in failure to properly align the strands [134]. Aberrant DNA morphology, including the presence of abasic sites and DNA adducts, can further stabilise the TOP1-DNA complex, creating DNA lesions [135]. Topoisomerase 2 (TOP2) enzymes resolve topological problems by a "two-gate" mechanism involving the hydrolysis of ATP [136]. They primarily induce DNA DSBs, but they can also induce SSBs [137] and oxidative DNA damage [137]. Three TOPs have been identified in mitochondria which are required by mtDNA. Furthermore, redox regulation of these TOPs may play a role in mitochondrial homeostasis [138].

#### DNA methylation

DNA methylation is an epigenetic process by which methyl groups are added to DNA, and it occurs most commonly to the C base, forming 5-methylcytosine, although it can also be added to G and A, resulting in the formation of N7-methylguanine, N3-methyladenine and O6-methylguanine [20]. DNA methylation regulates gene expression and therefore is a normal endogenous process, but it can also result in DNA damage. The endogenous production of choline, betaine and nitrosated bile salts, as well as exogenous factors such as smoking, diet, pollution and N-nitroso compounds, can induce DNA methylation [101]. The C base can be modified by oxidation, forming 5,6-dihydroxycytosine [139], which is necessary for DNA de-methylation [140]. This can result in base transition mutations, development of abasic sites and minor methyl lesions on DNA [20]. However, failure to remove these methyl groups induces DNA damage [20]. The BER pathway repairs these lesions by cleaving the glycosylic bonds of methylated bases.

#### Cross-linking DNA damage

Crosslinks of DNA are produced when two nucleotides form a covalent linkage, and it can be either intra-strand, within the same strand, or inter-strand, between opposite strands. Whereas intra-strand crosslinks are easily removed by NER, ICLs are extremely toxic lesions that prevent separation of the DNA strands, and as few as 20 unrepaired ICLs can kill mammalian cells [141]. ICL can be induced both by UVA and by chemical agents, including those used in chemotherapy, such as carboplatin and mitomycin C (MMC) [142]. Processes such as replication and transcription, where separation of the two DNA strands is essential, are inhibited by the presence of this irreversible covalent linkage, which can induce cell death [143]. The formation of ICLs requires two independent groups in an alkylating molecule that react with two bases present on opposite DNA strands. Oxidative stress and agents such as platinum compounds, MMC, psoralens, and nitrogen mustards induce ICLs [143]. ICLs are repaired by NER and other mechanisms [144].

#### DNA damage induced by lipid peroxidation

One of the consequences of excessive amounts of ROS and RNS is lipid peroxidation [145], whereby oxidants attack lipids containing one or more carbon–carbon double bonds [146]. This can induce DNA damage by the formation of reactive aldehydes, which produces mutagenic adducts in bases, particularly A and G [147, 148]. Among lipids, cholesterol esters, phospholipids, and triglycerides are particularly susceptible to oxidative modification because they contain polyunsaturated fatty acid (PUFA) side chains [30]. These PUFAs are extremely vulnerable to oxidation by free radical species, especially ^·^OH. Aldehyde products resulting from lipid peroxidation include 4-hydroperoxy-2-nonenal (4HNE) [149, 150], malondialdehyde (MDA) [151], and acrolein [152]. The oxidation of lipids is an important source of DNA damage [153, 154] and the reader is referred to several excellent reviews for more details [9, 155, 156].

## DDR and DNA repair pathways

The DDR is an elaborate signalling network that detects, signals and repairs DNA lesions [20]. Specific DNA repair pathways are activated based on the type of damage induced [21, 157, 158], regulated by phosphatidylinositol 3-kinase (PI3K)-like kinase family members. These include ataxia telangiectasia mutated kinase (ATM) and ataxia telangiectasia and Rad3-related protein (ATR), that mediate DSB and SSB repair, respectively [159]. In dividing cells, the DDR arrests the cell cycle following DNA damage, both transiently by activating DNA damage checkpoints, and permanently by inducing cellular senescence [160]. Below we discuss the DNA repair pathways relevant to redox-relevant DNA damage.

### BER pathway

BER is the most important mechanism that cells use to repair lesions formed following oxidative stress and redox dysregulation. It corrects damage to bases resulting from deamination, oxidation, or methylation, that have not significantly altered the arrangement of the DNA helix [161–163]. The nucleus is the main subcellular location where BER takes place, although it has also been detected in mitochondria [163].

DNA glycosylases play important roles in BER because they both detect and remove specific damaged or inappropriate bases, forming abasic sites whilst leaving the sugar phosphate backbone intact. At least 11 distinct types are involved in BER and 8-oxoG glycosylase (OGG1) initiates repair of the most common 8-oxoG lesions in both the nucleus and mitochondria [164]. The abasic site is then repaired by either ‘short-patch’ BER or ‘long-patch’ BER. In short-patch BER, which involves only a single nucleotide gap in the abasic site, DNA polymerase β (which is specific to BER) fills this gap, accompanied by the XRCC1/Ligase III complex [21]. In contrast, long-patch BER involves a repair tract of at least two (and up to 13) nucleotides, where the gap is sealed by Ligase I or proliferating cell nuclear antigen (PCNA) after resynthesis of DNA [144, 165]. BER is also implicated in the repair of SSBs through single-strand break repair (SSBR) [144]. Replication protein A (RPA) is required for each of the four major DNA repair pathways [166].

APE1 performs an important role in BER by acting as a nuclease, precisely cleaving the DNA backbone at the abasic site. During this process, APE1 is multifunctional because it displays endonuclease, 3′ phosphodiesterase, 3ʹ-to-5ʹ exonuclease, and RNA cleavage activities. Importantly, the exonuclease activity is required to remove DNA damage generated by ROS during oxidative stress, hence it is an essential component of BER. Moreover, APE1 forms a central link between redox regulation and DNA repair because it is the only DDR protein that can also regulate redox conditions. Hence it possesses two functions within the one protein (mediated by different domains), and thus it is also referred to as ‘redox effector factor 1,’ or ‘Ref-1’. APE1 also regulates multiple redox-regulated transcription factors, including nuclear factor kappa B (NF-κB) [167], STAT3, p53 [168], hypoxia inducible factor-1α [169], and cAMP-response element binding protein [169] (see also Sect. "AD" below).

### NER pathway

NER is the central pathway responsible for the removal of large ssDNA adducts induced by UV irradiation, environmental mutagens, or chemotherapeutic agents [144, 170]. Moreover, NER also repairs lesions that result from oxidative stress [171]. There are two sub-pathways of NER: global genome NER (GG-NER) and transcription-coupled NER (TC-NER) [172] that differ in how they are initiated.

**Fig. 4:**
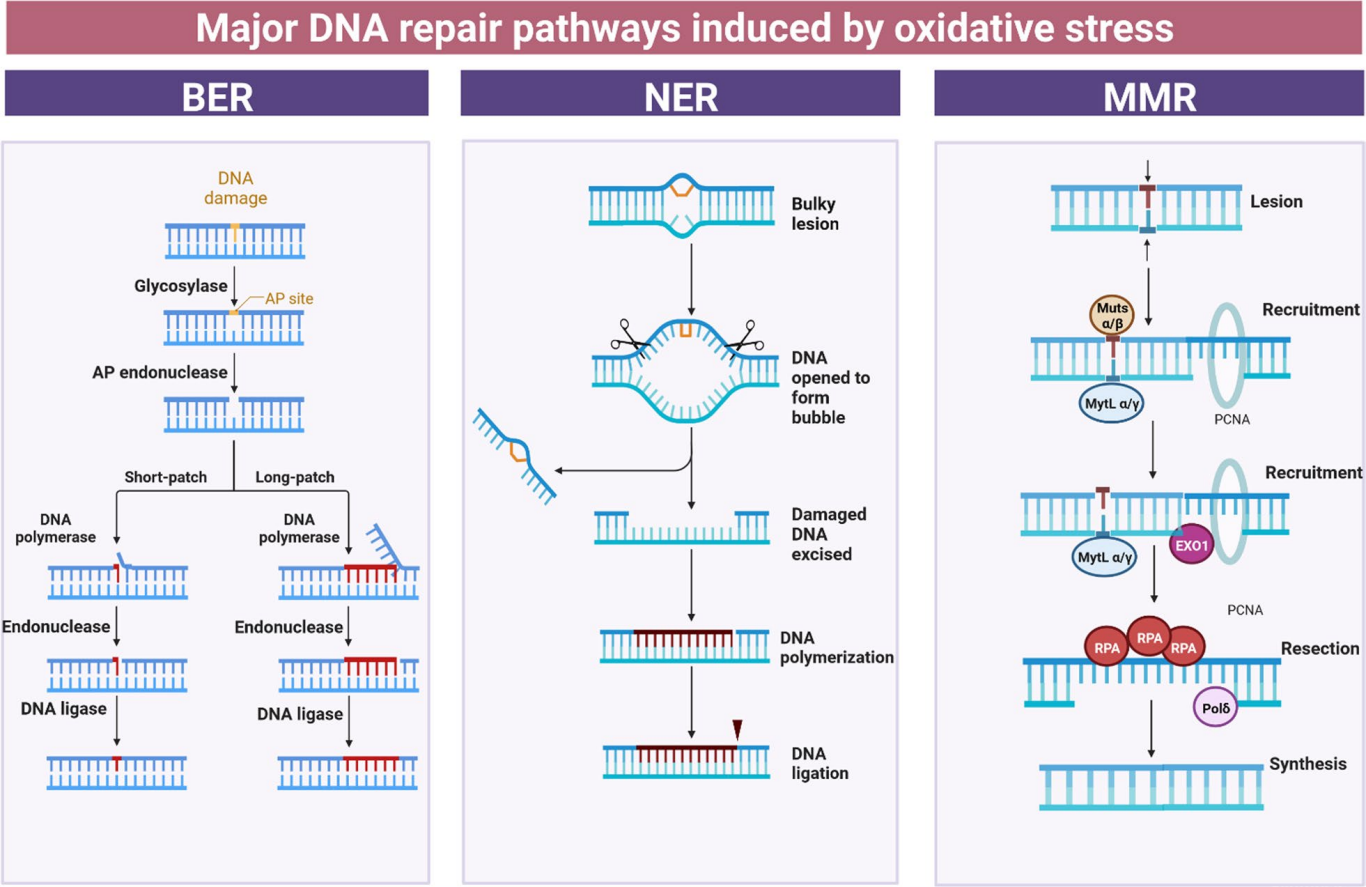
The major DNA repair pathways involved in correcting DNA damage induced by oxidative stress. BER is the predominant repair mechanism that removes oxidative DNA damage to bases, via two general pathways—short-patch and long-patch. Short-patch BER facilitates repair of a single nucleotide, whereas long-patch BER repairs two or more nucleotides. Although the nucleus is the main subcellular localisation where BER takes place, it has also been detected in mitochondria [163]. NER is the principal pathway responsible for the removal of large single-stranded DNA adducts induced by UV irradiation, environmental mutagens, or chemotherapeutic agents, but it is also implicated in repairing oxidative DNA damage. MMR is another pathway that repairs DNA damage induced by oxidative stress. MMR is responsible for the detection and repair of errors produced during DNA replication, involving the incorrect insertion, deletion or misincorporation of nucleotides. *RPA* Replication protein A; *pol δ* DNA polymerase δ; *Exo1* exonuclease 1

Unlike TC-NER, GG-NER is not induced during transcription. Specific proteins continuously scan the genome for distortions of the helix. Once detected, GG-NER is then initiated by either a complex of xeroderma pigmentosum complementation group C (XPC) and UV excision repair protein radiation sensitive 23 B (RAD23B) (XPC-RAD23B) alone, or in some cases, with UV-damaged DNA-binding protein [173]. In contrast, TC-NER is activated during transcription when RNA polymerase is stalled at a lesion with TC-NER specific proteins: Cockayne syndrome protein A, CSB, and XPA-binding protein 2. Once the lesion is identified, both TC-NER and GG-NER follow a similar mechanism, requiring transcription factor II H to excise and repair the lesion [170]. Bi-directional helicase reveals approximately 30 nucleotides in DNA during this process, stabilised by XPA and RPA. The lesion is removed by XPG and the DNA excision repair protein-1 (ERCC1)-XPF complex, which leaves a single-stranded gap. This is filled in by DNA replication proteins PCNA, RPA and DNA polymerases POL σ, κ, ε, and subsequently sealed by DNA ligases I or III [144]. The NER pathway is also involved in the early steps of ICL repair [144] (Fig. 4).

### Mismatch repair (MMR) pathway

The MMR pathway is responsible for the detection and repair of errors produced during DNA replication, involving the incorrect insertion, deletion or misincorporation of nucleotides. This prevents the permanent incorporation of mutations in dividing cells.

The MMR pathway comprises three linked, but different, protein subunits in humans: hMutSα, hMutSβ, and hMutLα. hMutSα is a heterodimer composed of hMSH2 and hMSH6, and it is constantly expressed, scanning the homoduplex DNA for errors [174]. Once mismatched bases are recognized, MMR is activated through upregulation of MSH2 and hMSH6. This complex then interacts with component proteins of the hMutLα heterodimer, MLH1 and PMS2, resulting in its binding to DNA lesions [165] to facilitate removal of the mismatched bases. RPA binds to nicked heteroduplex DNA to facilitate assembly of the MMR initiation complex [166]. Finally, the new DNA is synthesized (Fig. 4).

### ICL repair pathway

ICL repair is highly conserved, and it maintains genomic stability during DNA replication. Two mechanisms are present in humans, recombination-dependent and recombination-independent pathways. Recombination-dependent ICL involves fanconi anemia (FA) proteins both in its detection and repair. FA is an autosomal recessive cancer syndrome characterized by progressive bone marrow failure and congenital anomalies, involving eight different complementation groups. Several FA proteins interact in a multi-subunit complex that repairs complex ICL lesions in DNA. The formation of an ICL prevents the DNA strand from unwinding and separating, which stalls the replication fork. A complex containing FA proteins FANCM and FAAP24 as well as the histone fold protein complex, binds to the stalled replication fork, which is then remodelled. This results in migration of the Holiday junction, unwinding of ssDNA [20], and activation of ATR-mediated Checkpoint kinase 1. This further activates FA proteins FANCI and FANCD2, which together form the ‘ID complex’. Phosphorylation of this complex by ATR results in its recruitment to the FA core where it becomes mono-ubiquitinated, and this subsequently recruits other DDR factors involved in ICL repair. Other DNA repair pathways, including homologous recombination, NER and translesion DNA synthesis, also contribute to the repair of ICL [22]. Failure to repair ICLs results in significant chromosomal abnormalities that promote tumorigenesis [175]. Moreover, following oxidative stress, ICLs can be generated. This has been reported for thymine radicals [176] and another major oxidative lesion (to A bases), oxoA, which can produce ICLs with the opposite base (G, A, C, T, I), to produce structurally diverse ICLs [177]. However, this mechanism has not been described for the most common oxidative lesion, 8-oxoG [177].

### SSBR pathway

SSBs can arise directly, by breakdown of oxidized sugars or indirectly, through BER of oxidized bases, abasic sites or damaged/modified bases [161, 178, 179]. They pose severe risks to genetic stability and cell viability if not repaired promptly [81]. Therefore, cells have developed efficient mechanisms to repair SSBs, collectively known as SSBR [81], which is often considered to be a component of BER [180]. The overall SSBR process comprises four fundamental steps, involving SSB detection by ATR, DNA end processing, DNA gap filling and DNA ligation [81]. This also provides another mechanism to repair ROS-induced DNA damage, but its role is not as significant as BER, NER and MMR.

A growing body of evidence indicates that poly-ADP-ribose-polymerases (PARPs) perform a central function in SSBR [181–184]. The PARP enzyme family (consisting of 17 members) catalyzes the covalent transfer of polymers of ADP-ribose onto acidic residues in target proteins, using the redox substrate NAD + . This process, termed ‘PARylation’, is a post-translational modification that regulates multiple signalling mechanisms, including several DNA repair pathways. PARP1 and PARP2 are induced instantly after the formation of SSBs, which results in PARylation of DNA ligase III, DNA polymerase beta, XRCC1, and end-processing enzymes such as TDP1 and aprataxin, at sites of SSB damage [21]. Recruitment of chromatin remodelling factor ALC1 (amplified in liver cancer 1) and macroH2A1 at DNA damage sites is also PAR-dependent [22]. This leads to chromatin remodelling and the recruitment of complexes to facilitate DNA repair and chromatin modification [22]. Finally, DNA polymerase β, polynucleotide kinase (PNK) and nucleases APE1, aprataxin PNK-like factor (APLF) and aprataxin (APTX), seal the processed DNA [21]. The activation of PARP-1 following DNA damage and subsequent depletion of NAD + and then ATP, have been linked to energy failure, redox dysregulation, mitochondrial ROS production and apoptosis [185].

### DSB repair pathway

There are two main pathways to repair DSBs in eukaryotes: non-homologous end joining (NHEJ) or homology directed repair (HDR) [26, 186, 187]. However, in terminally differentiated neurons, NHEJ is thought to be the most important mechanism [188] because HDR requires the presence of homologous sequences during the S and G2/M phases of the cell cycle, which is absent in neurons. Hence HDR functions only in the S-phase in undifferentiated or proliferating and neuronal stem/progenitor cells, although genome editing via HDR is possible in mature post-mitotic neurons [189]. Even low levels of ROS are known to produce complex DSBs and can produce mutations following error-prone NHEJ [189].

#### ATM-mediated DSB repair pathway

In both the homologous recombination and NHEJ pathways, DSBs are initially recognised by ATM. As for SSBR, activated PARP family members (PARP1 and PARP2) are rapidly recruited and bind to the MRN/ATM complex at DSBs [22]. This leads to recruitment of other DDR proteins [22] and activation of ATM. H2A histone family member X (H2AX) is then phosphorylated by ATM over a large region of DNA surrounding a DSB. Phosphorylated H2AX (γH2AX) forms visible foci in the nucleus that are widely used as an experimental marker of DSBs. Furthermore, γH2AX possesses important functions in the DDR by (i) assembling key substrates of ATM at sites containing damaged chromatin, including p53, p53-binding protein, and breast cancer gene 1 (BRCA1), and (ii) activating checkpoint proteins that arrest the cell cycle, such as Mdc1 (mediator of DNA damage checkpoint protein 1) and Chk2 [22, 190].

#### NHEJ pathway

There are two main mechanisms of NHEJ. Classical NHEJ results in ligation of DNA ends with no or little (1–3 bases) sequence homology, thus it can result in the production of deletion or insertion mutations [191]. In contrast, in alternative NHEJ, regions of DNA with micro-homologous sequences are ligated (4–20 bases) [191]. The molecular mechanisms involved in alternative NHEJ remain unclear, although it is thought to be a back-up system that is less efficient than classical NEHJ [192]. However alternative NHEJ has an even greater tendency to create mutations because the ligated products always contain deletions [192].

#### Classical NHEJ

Neurons remain arrested in the G0 phase of the cell cycle, but NHEJ repairs DNA throughout all phases of the cycle. NHEJ involves the detection of DNA ends, assembly and stabilization of the NHEJ complex, linking and then processing of the ends, and finally, attachment of the ends and disassembly of the NHEJ complex [193]. A specific kinase complex, DNA protein kinase (DNA-PK, another PIKK family member), performs a critical role in NHEJ. It consists of Ku, a heterodimer of Ku70 and Ku80 subunits, which directs the complex to DNA and activates the PI3K kinase activity of its catalytic subunit (DNA-PKcs). Upon DNA damage, Ku (Ku70/Ku80) creates a ring-like structure and rapidly binds to DNA strands at DSB sites [22]. Ku also facilitates the threading of PARP1 onto each broken DNA end and DNA end processing, and it also recruits nucleases to trim, and polymerases to fill in, the DNA ends [26]. DNA-PKcs substrates, such as X-ray repair cross-complementing protein 4 (XRCC4), become phosphorylated upon its activation, resulting in DNA end protection. Ultimately, DNA ligase IV complex and Cernunnos/XLF then ligate the released DNA ends [144]. However, nucleases ARTEMIS and APLF, along with PNK kinase/phosphatase, are responsible for processing the DNA ends that cannot be ligated [144]. In a small proportion of NHEJ (10%) repair events, ARTEMIS is phosphorylated by ATM, even though it is considered to be a DNA-PKcs substrate [144].

#### Alternative NHEJ

Whilst this end-joining mechanism is not well characterised, it does not involve the same machinery as classical NHEJ. Alternative NHEJ can result following primary DNA re-section by PARP and MRN when Ku or recombination factors are not available, and it requires the DNA end-processing factor, CtBP-interacting protein (CtIP). DSB breaks are sealed by microhomology-mediated base-pairing, followed by nucleolytic trimming of DNA flaps, DNA gap filling, and then ligation. In alternative NHEJ, XRCC1 and LIG III protect and ligate the DNA ends, respectively [22]. There is evidence that DNA polymerase θ is of critical importance for this mechanism [26].

#### Homologous recombination pathway

Homologous recombination is the most common form of HDR used to repair DSBs and ICLs. It uses a homologous sequence as a DNA template to repair the DSB, resulting in reformation of the original DNA sequence. Hence, it is less likely to introduce mutations compared to NHEJ. Following generation of the DSB, the MRN complex, consisting of MRE11, Rad50 and Nbs1, binds to DNA on either side of the DSB. Rad51 becomes recruited into DSB sites following activation of ATM by MRN [22]. DNA end resection follows, whereby the dsDNA is processed by the MRN complex, CtIP, PARP1, BRCA1 and other endonucleases, to remove nucleotides from the 5' end to produce short 3′ single-strands (30 bases) [22, 144]. This also prevents activation of classical NHEJ. MRE11 stabilizes the ends of DNA, and it facilitates the early steps of DNA end resection via its endonuclease and exonuclease activities [22, 194]. The two other members of the MRN complex, Rad50 and NBS1, promptly interact with MRE11 at the DNA ends of the DSBs and NBS1 binds to ATM by its C-terminal domain, which accelerates its recruitment to DSB sites [22]. RPA, which possesses a high affinity for ssDNA, binds to the 3’ ssDNA overhang and Rad51 then binds, forming a nucleoprotein filament. Rad51, aided by BRCA2, searches for a homologous sequence to the 3’ overhang on the sister chromatid. Once located, the nucleoprotein filament catalyses invasion of the strand. Base pairs on the homologous DNA strands are consecutively exchanged at specific regions, that move the branch point up/down the DNA sequence in a process called ‘branch migration’[195]. These regions are termed ‘Holliday junctions’, which contain four double-stranded DNA arms joined in a branched structure. The DNA strands with DSBs are thus replaced by a homologous sequence template on the sister chromatid [196].

#### Single-strand annealing (SSA)

SSA is another DSB repair pathway that is activated when a DSB occurs between two repeated sequences oriented in the same direction. It is considered to be midway between homologous recombination and NHEJ because it uses homologous repeats to bridge the DSB ends [196]. Next to the DSB, ssDNA regions are formed that extend to the repeat sequences so that complementary strands can anneal to each other. The annealed sequences are then processed by digestion of the ssDNA overhangs and filling in of the gaps. SSA plays a role in MRN exonuclease-mediated DNA end excision from both strands of the DSB site, until small homologous sequences on both strands are established [197].

## Redox regulation, DNA repair and DNA damage

Increasing evidence indicates that the DDR is regulated by the cellular redox state, and vice versa. Furthermore, redox regulation also controls epigenetic mechanisms and other mechanisms related to DNA damage, such as the regulation of gene expression by transcription factors. As detailed below, there are several known links between redox regulation and maintenance of DNA repair mechanisms in normal cellular physiology, although some aspects are only just emerging. Below we provide an overview of these mechanisms in normal cells.

### Proteins with possible dual functions in both DNA repair and redox regulation

APE1 is the classic example, given it possesses both redox activity and DNA repair functions in the nucleus [198], and it is protective following oxidative DNA damage to neurons. It functions as a redox effector factor for multiple transcription factors including AP1, p53 and HIF1-α. In addition to its well-established role in BER, the redox activity of APE1 is also implicated in NER [198, 199]. Similarly, whilst Ku is an essential component of NHEJ [200], it also possesses redox activity, which is essential for its binding to DNA [200]. The ability of Ku to bind to DNA damage sites decreases following induction of oxidative stress [200].

O-6-methylguanine-DNA methyltransferase (MGMT), which facilitates chemoresistance to alkylating agents, is one of the most specific DNA repair enzymes, and it is also redox-regulated [201]. MGMT replaces the highly mutagenic lesion O6-methylguanine with guanine, thus inhibiting mismatch errors and other mistakes that arise during transcription and DNA replication. During this process, MGMT transfers the methyl at O6 sites of damaged guanine nucleotides to cysteine residues [201]. S-nitrosylation at these active, essential cysteines can inhibit its enzymatic activity through a ‘suicide’ reaction [202]. Therefore, inhibition of MGMT can result following induction of oxidative stress [203].

In addition to direct effects on the DDR, redox regulation can modulate gene expression of DNA repair proteins [203]. Transcription factors with zinc-finger motifs or transition metal-binding regions are regulated by redox processes [204]. Both the Trx and Grx systems are upregulated by Nrf2, a transcription factor with a zinc-finger that controls many genes displaying antioxidant response elements and is a master regulator of the antioxidant response. Nrf2 is in turn regulated by thiol oxidation of kelch-like ECH-associated protein 1 [205].

Another zinc-binding transcription factor with multiple important roles in the DDR is p53 [206]. p53 is a multi-tasking protein activated by DNA damage that co-ordinates several DNA repair activities. Thus, it has been termed a “guardian of the genome” [207]. p53 participates in several DNA repair mechanisms and it controls DNA-damage checkpoints by halting the cell cycle, to allow time for repair. Cells lacking p53 are prone to mutations and genomic instability, hence it is also known as a tumor suppressor. Moreover, p53 is regulated by redox signalling, thus it has been referred to as a central ‘hub’ in redox homeostasis [208] because it also mediates antioxidant and pro-oxidant pathways [208]. In addition, it protects neurons from DNA-damaging agents [209]. Importantly, its function as a transcription factor and its ability to bind to DNA are dependent on cellular redox conditions. p53 contains 10 cysteine residues located in its DNA-binding region, three of which form the zinc fingers that are crucial for its correct folding. APE1 stimulates p53-dependent transcription [207, 210] and p53 activity is dependent on the Trx system, both directly and indirectly via APE1. The crosstalk between Trx and p53 involves TXNIP, which interacts with p53. TXNIP binds to and stabilises p53, and it detaches from Trx during oxidative stress. S-glutathionylation of cysteines in human p53 inhibits its DNA-binding property [211]. p53 also activates expression of several antioxidant genes, including sestrin (*Sesn*)*1* and *Sesn2* [212]. Interestingly, promoters of p53-regulated genes with antioxidant functions appear to be sensitive to low levels of p53, whereas pro-oxidant and pro-apoptotic p53 target genes are activated in response to higher p53 levels upon extensive stress [211].

There is increasing evidence that PDI proteins have a role in the DDR, particularly Erp57. Inhibition or knockdown of PDI family members (PDIA1 and PDIp) downregulates many DNA repair genes, including E2F transcription factor 1 and Rad51 [213, 214]. Similarly, Erp57 binds to DNA [215] and PDI/Erp57 immunoprecipitates with APE1 [41]. In addition, Erp57 can translocate into the nucleus where it binds to MSH6 and DNA polymerase δ, following oxidative stress [216]. ERp57, together with high-mobility group proteins 1 and 2 (HMGB1 and HMGB2), is part of a complex that recognises damaged DNA [217]. Erp57 also modulates phosphorylation of H2AX, and it relocates to the nucleus following DNA damage [218]. Downregulation of Erp57 significantly inhibits the phosphorylation of H2AX and induction of DDR following cytarabine treatment [218]. However, despite this evidence, a direct role for PDI proteins in DNA repair has not yet been described.

SOD1 is an important antioxidant protein expressed in the nucleus, as well as the cytoplasm and mitochondria [219–221], and recently a protective role for nuclear SOD1 against oxidative DNA damage was described [222]. Following oxidative stress, SOD1 is phosphorylated by Chk2, leading to its translocation to the nucleus and protection against DNA damage [222]. Nuclear SOD1 appears to regulate the expression of GSH during this process [223]. The nuclear localisation of SOD1 is enhanced in an ATM-dependent manner by its association with the Mec1/ATM effector, Dun1/Cds1 kinase, and phosphorylation of SOD1 at S60 and S99 [222]. Ribonucleotide reductase is a SOD1 transcriptional target [224] vital for the synthesis of deoxyribonucleosides and hence essential for DNA repair [225]. In addition, recent studies have shown that ATM also has a redox-sensor function in mitochondria, and can regulate oxidative stress by this mechanism.

## Redox dysregulation and DNA repair in neurodegenerative diseases

The basic molecular mechanisms linking redox regulation to DNA damage remain unclear. However, improving our understanding of how these events are inter-related has important implications for diseases in which both processes are implicated in pathogenesis, such as neurodegenerative conditions [203].

There is now significant evidence linking defects in the DDR to neurodegenerative diseases [17] and the death of specific types of neurons is the underlying pathological feature of these conditions. However, the mechanisms by which neurons die in these conditions remain unclear, although it is well established that apoptosis plays a role [23]. Recently, many novel cell death pathways have been identified, and the Nomenclature Committee on Cell Death (NCDD) has developed guidelines to describe these mechanisms [226]. Importantly, several of these processes are induced by DNA damage, and there is increasing evidence that neurons die by at least some of these mechanisms. Parthanatos is a PARP-1-dependent cell death mechanism distinct from apoptosis or necrosis that can be induced by oxidative stress and DNA damage. It involves overactivation of PARP-1 leading to augmented production of long-chained and branched PAR polymers [23]. Importantly, multiple reports have implicated parthanatos in the death of neurons in several neurodegenerative diseases. Similarly, a role for p53 in neuronal death in neurodegenerative disorders has also been reported in several studies. For more details of these studies, and cell death mechanisms in relation to DNA damage in neurodegenerative disorders, please see a recent review [23].

It is also important to consider the role of glial cells in relation to oxidative DNA damage, neuroinflammation and neurodegeneration. When activated, microglia can produce several factors that are toxic to neurons, such as pro-inflammatory cytokines TNFα, PGE_2_, and INFγ and ROS (NO, H_2_O_2_, O_2_^·−^, NOO^−^), in response to diverse stimuli, including neuronal damage, misfolded proteins, and environmental toxins [227]. Microglial NOX2 is a major regulator of neurotoxicity by producing excessive ROS [228]. Through a complex antioxidant response, astrocytes also enhance the decomposition and clearance of free radicals produced by neurons and other cell types in the CNS [229]. Excessive free radicals can result in reactive astrogliosis, inducing neuroinflammation, which can lead to further oxidative stress [229]. Senescence is also strongly linked to DNA damage, and it is also implicated as a potential driver of neuroinflammation in neurodegenerative diseases [20].

Various environmental stressors are implicated as risk factors for neurodegenerative diseases, and interestingly they are also involved in aging, oxidative stress and/or DNA damage [230]. Of these, heavy metals (Pb, Cd, As, Hg, Cu, Zn and Fe) and pesticides (1-methyl-4-phenyl-1,2,3,6-tetrahydropyridine [MPTP], paraquat, dieldrin, rotenone [231]) in particular are associated with neurotoxicity and induction of DNA damage via oxidative stress [232].

In the next part of the review, we will focus on neurodegenerative disorders and evidence linking redox dysregulation and DNA damage in each condition. Many of the relevant studies have employed animal models or murine cell lines. However, whilst these reports have provided interesting insights into the role of the DDR in these neurodegenerative diseases, it is important to also consider that there may be species differences in DNA repair mechanisms between mouse and human neurons [233, 234]. Human and mouse neurons possess different DNA repair kinetics and respond differently to oxidative stress. Furthermore, following DNA damage, differences in cell death, chromatin condensation, and activation of DDR sensor proteins are also evident [234].

### AD

AD is the most common neurodegenerative disease [235]. It is characterised clinically by progressive memory loss with neuropsychiatric symptoms due to the degeneration of cortical neurons in the entorhinal cortex and hippocampus [236]. The pathological hallmarks of AD include the accumulation of cytoplasmic senile plaques composed of amyloid beta (Aβ) peptides (resulting from cleavage products of amyloid precursor protein) and the formation of neurofibrillary tangles (composed of hyper-phosphorylated tau) [237, 238]. Most AD cases are sporadic in nature. However, 5%–10% cases are familial with a predominately autosomal dominance inheritance pattern, consistent with polygenic origins and multifactorial pathogenic disease processes [239]. Several mechanisms have been implicated in the pathogenesis, including both oxidative stress and DNA damage [236, 240].

There is direct evidence linking redox dysregulation with DNA damage in AD. Increased levels (twofold) of DNA strand breaks were observed in the cerebral cortex of AD brains [241]. Higher levels of oxidative DNA damage, in the form of 8-OHG adducts and oxidized purine and pyrimidine bases, were detected in peripheral leukocytes of AD and mild cognitive impairment (MCI) patients compared to healthy controls [242–244]. Similar observations were made in AD lymphocytes [245]. Another study reported an age-dependent increase in the levels of 8-OHG in DNA in the cerebral cortex of AD patients. Consistent with this finding, elevated levels of 8-OHG, 8-hydroxyadenine (8-OHA) and 5-hydroxycytosine were detected in the total DNA of AD parietal lobe regions compared to matched controls [245]. The same study also observed higher levels of thymine glycol, 5-hydroxyuracil, 4,6-diamino-5-formamido-pyrimidine (FapyAde), and FaPyGua in several AD brain areas. In another study, significantly higher levels of 8-OHG, 8-OHA and 5-OHU were detected in the temporal and parietal lobes of AD compared to control patients [246]. Expression of adducts 8-OHG in RNA and 8-hydroxy-2’-deoxyguanosine (8-OHdG) in DNA, were also observed in late-stage AD compared to age-matched control patients [247].

Significantly more aldehydic by-products HNE and acrolein were detected in late-stage AD brains and CSF, including in the most vulnerable areas (hippocampus and superior and middle temporal gyri) of MCI and early-stage AD brains [248]. Moreover, higher levels of acrolein/guanosine adducts were also observed in the hippocampus of late-stage AD patients compared to controls [249]. Mutations in the gene encoding OGG1 have been identified in AD patients, resulting in reduced enzymatic activity [250]. Reduced levels of OGG are present in AD brains, implying that BER is defective in affected neurons [251]. Consistent with this notion, defective BER, diminished activity of DNA glycosylase and reduced DNA synthesis by DNA polymerase β have been reported in AD tissues [252].

More excision repair cross-complementing gene products have been also identified in AD brains compared to controls, suggesting that DNA repair pathways are activated to counteract increased oxidative damage [253]. In addition, higher levels of SSBs and small increases in DSBs were observed in AD brains [254]. In contrast, reduced DNA repair of SSBs [255] or DSBs by DNA-PK-mediated NHEJ [256] were reported in AD brains compared to controls. Similarly, significantly low levels of MRE11 DNA repair complex proteins were identified in the neocortex of AD brains [257]. This would hamper the recognition of DNA damage and its subsequent repair, contributing to neuronal death in AD [248].

Impaired SOD1 activity has also been detected in AD animal models and post-mortem AD brains [258]. The expression of SOD1 and SOD2 is elevated in age-matched AD brain tissues compared to controls [259]; however, the activity of both enzymes decreases significantly in the same tissues [259]. Enhanced formation of Aβ plaques, neuroinflammation, tau phosphorylation, and consequent memory decline have also been observed in SOD1-deficient Tg2576 mice [258, 260].

Oxidative imbalance and mitochondrial dysfunction are observed in AD [261–263], together with oxidative stress-induced mtDNA damage. Significantly higher levels of 8-OHdG and 8-OHG were reported in AD brains compared to age-matched control samples [264–266]. Another study analysing oxidized nucleosides revealed three-fold more oxidative damage in mtDNA in AD patients [266]. In addition, sporadic mutations were detected in mtDNA of AD brain tissues [267, 268]. Similarly, mutations in mtDNA in the blood of AD patients and in the lymphoblastoid lines derived from the blood of AD patients have been reported [269].

Elevated levels of both PAR polymers and PARP-1 were detected in neurons of human AD brain tissues [270, 271]. In addition, overexpression of PARP-1 is observed in AD brains, largely in the frontal and temporal lobes [240], and the accumulation of Aβ peptides is preceded by oxidative stress and upregulation of PARP-1 in the hippocampus of adult rats [272]. Similarly, a study in SHSY-5Y cells revealed that upregulation of PARP-1 induces pathological features of AD such as deposition of Aβ and the formation of tau tangles [273]. Moreover, co-immunoreactivity of PARP/PAR with Aβ, tau and microtubule-associated protein 2 has been observed in human AD brain tissues [274, 275]. p53 is increased in the temporal cortex of AD patients [276, 277]. Expression of Aβ peptides triggers p53-mediated microglial apoptosis and microglial neurotoxicity [278]. p53 is also prone to aggregate and is a component of misfolded aggregates in a tau mouse model and in human AD brains [279]. Interestingly, p53-mediated DDR has been found to be impaired in AD [279]. Taken together, these studies provide evidence that redox imbalance is associated with DNA damage and inefficient DNA repair, which together contribute to neurodegeneration in AD.

### PD

PD is the second most common neurodegenerative disorder [11]. It is characterised by the loss of dopaminergic neurons in the substantia nigra pars compacta (SNpc) accompanied by the formation of intraneuronal inclusions called Lewy bodies [11, 280, 281]. The majority of PD cases (95%) are sporadic while only 5% of cases are linked to mutations in specific genes [11]. Multiple lines of evidence implicate both oxidative stress and DNA damage as key mechanisms in disease pathogenesis [282–285].

ROS-induced DNA damage in the form of oxidized bases and impaired repair of SSBs has been implicated in PD etiology [84]. Studies have reported elevated levels of 8-OHdG, resulting from DNA oxidation, in PD brains [11, 282] and increased levels of 8-OHG in the SNpc of PD patients [282]. Similarly, more DNA damage, indicated by elevated levels of markers γH2AX and p53-binding protein foci, is present in dopaminergic neurons of two synucleinopathy PD mouse models [286]. Further in vitro studies with dopaminergic SH-SY5Y cell lines suggested that excessive oxidation is at least partially responsible for DDR activation observed in vivo [286]. In comparison to age-matched controls, the SNpc of PD patients displays increased SOD levels, whereas the activities of CAT, GPx and GR are similar as controls [287]. Reduced levels of GSH and altered GSH/GSSG ratio, resulting in more of the oxidized form, have been detected in the SNpc of PD brains [288]. Similarly, depletion of GSH is observed in patients with a pre-symptomatic form of PD, known as incidental Lewy body disease, compared to control subjects [289]. Under elevated oxidative stress conditions, reduction in GSH results in dopaminergic neuronal loss [290]. In addition, depletion of GSH results in increased NO and MPTP/MPP + toxicity in dopaminergic neurons in animal models of PD [291, 292]. Glutamyl cysteine ethyl ester and GSH ethyl ester, two precursors of GSH, increase GSH levels in neuronal cells both in vitro and in vivo and are protective against oxidative and nitrosative stress [293, 294]. Similarly, intracellular GSH levels are also rescued by thiol antioxidants such as α-lipoic acid in both in vitro and in vivo PD models [295, 296]. Depletion of the antioxidant vitamin C has also been detected in PD [297] and vitamin C levels in lymphocytes may be a potential biomarker of disease progression in PD [298]. Furthermore, cells with lower levels of uric acid (UA) are more vulnerable to oxidative damage [299] and individuals with low cellular uric acid levels may be at a greater risk of developing PD [300]. UA prevents 6-hydroxydopamine (6-OHDA)-induced oxidative damage in neuron-like PC12 cells and increases GSH and SOD1 [301]. Similarly, GSH levels, SOD1 activity and dopaminergic neuronal damage are rescued in a 6-OHDA rat model of PD following UA treatment [302]. SOD1 may be a first-line protection against enhanced ROS production in PD patients [303]. RNS, such as NO and its metabolite PN, may also cause DNA damage in PD [11] by reacting with superoxide anion radicals. NO can then generate more oxidatively active PN, which in turn may induce DNA fragmentation [74].

Recent studies have identified mtDNA damage in PD [304] and abasic sites in mtDNA of dopaminergic neurons in PD post-mortem human and rat brains [305], which precede the onset of neurodegeneration [305]. Significant accumulation of abasic sites in dopaminergic neurons, but not in cortical neurons, has been detected [305–307]. Elevated levels of ROS render dopaminergic neurons in the SNpc more prone to DNA damage and contribute to neurodegeneration [305, 308]. Consistent with this notion, BER activity is increased in the SNpc of PD patients [309–311]. Knockout mouse models lacking DNA repair enzymes (human MutT homolog 1, an oxidized purine nucleoside triphosphate; and OGG1) are more susceptible to dopaminergic toxins and age-related degeneration in the nigrostriatal system [312, 313]. Moreover, transgenic mouse models expressing a mitochondrial-targeted restriction enzyme causing mtDNA damage in dopaminergic neurons recapitulate many of the key features of PD, including motor phenotype, progressive loss of dopaminergic neurons in the SN and depletion of dopamine in the striatum [314]. Taken together, these studies imply that redox dysregulation can induce mtDNA damage in PD and may contribute to neurodegeneration. α-Synuclein is co-localized with γH2AX and PAR in human HAP1 cells and in transgenic α-synuclein mouse models [315]. Reducing α-synuclein levels using bleomycin results in higher DSBs and impaired DNA repair in these cells [315]. Moreover, α-synuclein knockout mice show increased DSB levels [315], suggesting that it may play a role in DNA repair. Interestingly, increased DNA damage and dopaminergic neuronal death have been observed in two PD mouse models [286].

P53-mediated selective cell death is also evident in PD. NO-induced, p53-mediated dopaminergic neuronal death has been observed in a mouse SNpc-derived cell line (SN4741) as well as in vivo models of PD [316]. The neurotoxin 6-OHDA is widely used to induce selective degeneration of dopaminergic and noradrenergic neurons and therefore, can imitate PD symptoms [23]. DNA damage induced by 6-OHDA treatment is linked to p53-mediated cell death of primary dopaminergic neurons [317]. Together, these lines of evidence suggest that DNA damage resulting from redox dysregulation may contribute to neurodegeneration in PD. Several studies also reported parthanatos in PD. MPTP induces neurodegeneration of dopaminergic neurons in SNpc, leading to PD symptoms [281]. Several studies have linked neurotoxicity of MPTP to parthanatos of dopaminergic neurons. MPTP treatment induces DNA fragmentation both in vivo and in vitro [318, 319]. Similarly, PARP upregulation-mediated toxicity to dopaminergic neurons is observed following MPTP administration in a mouse model [320] and inhibition of PARP significantly attenuates these toxic effects [321, 322]. Activation of PARP-1 and progressive loss of dopaminergic neurons by parthanatos have also been reported in a transgenic mouse model overexpressing aminoacyl tRNA synthase complex-interacting multifunctional protein 2, a parkin (E3 ubiquitin ligase) substrate [323]. MPTP-induced parthanatos requires neuronal NO synthase [320], suggesting a link between MPTP-induced PARP activation and subsequent ADP-ribose polymerisation as well as NO-induced DNA damage. Increased NO levels are also observed in nigral cells in PD [324, 325].

### ALS

ALS is a fatal, rapidly progressing neurodegenerative disorder that affects motor neurons in the brain, brainstem, and spinal cord [326]. It is clinically, genetically and pathologically linked to FTD, which manifests as frontotemporal lobar degeneration [327]. Variants in more than 40 genes cause ALS, most common of which are those encoding SOD1, chromosome 9 open reading frame 72 (C9orf72), TAR DNA-binding protein-43 (TDP-43) and fused in sarcoma (FUS), which are all linked to both sporadic and familial forms of disease [328].

DNA damage is now increasingly implicated as an important pathophysiological mechanism in ALS [329–331], particularly with the identification of both TDP-43 and FUS as proteins with normal cellular functions in DNA repair [332–334]. Elevated levels of oxidative DNA damage are consistently observed in both sporadic and familial ALS patients [335, 336]. Moreover, DNA damage is associated with redox dysregulation in ALS. Increased levels of 8-OHdG have been detected in the motor cortex of sporadic ALS patients, and in the spinal cords of both sporadic and familial ALS patients [337, 338]. Similarly, analysis of plasma, urine and CSF of ALS patients revealed increased levels of 8-OHdG [338]. High levels of 8-OHdG have also been reported in the SOD1^G93A^ transgenic mouse model [339]. Decreased levels of BER enzymes DNA polymerases α and β have been detected in motor neurons of SOD1^G93A^ mice [340]. Furthermore, decreased mitochondrial activity of OGG1 and increased 8-OHdG levels have been detected in spinal motor neurons of sporadic ALS patients, indicating that impairment of redox function, resulting in oxidative stress, disrupts DNA repair in the mitochondria [341]. In addition, a polymorphism in *OGG1*, resulting in the substitution of serine with cysteine (Ser326Cys), reduces DNA activity and is associated with increased risk of sporadic ALS [342]. The levels of a common mitochondrial DNA deletion mutation (mtDNA4977) encoding a subunit III of the redox enzyme cytochrome oxidase, involved in OXPHOS [343], are higher in Brodmann area 4 of primary motor cortices in sporadic ALS patients compared to controls [343]. Moreover, increased abasic sites are also detected in spinal motor neurons of ALS patients compared to controls [330]. Likewise, the levels of 8-OHdG are increased in cells expressing SOD1-G37R and SOD1-G85R compared to wild-type SOD1 [344]. A meta-analysis examining the levels of blood oxidative stress markers in ALS patients reported increased levels of 8-OHdG, MDA (the end product of lipid peroxidation) and AOPP (advanced oxidation protein product, a marker of protein oxidation), and reduced levels of GSH, compared to healthy controls, which all reflect both DNA damage and redox dysfunction in ALS [345]. Hence, these data imply that DNA damage is closely associated with ALS and is linked to redox dysregulation.

Similar to the other neurodegenerative diseases, PARP1 hyperactivation and toxicity are implicated in ALS pathogenesis [346, 347]. Elevated PAR levels are observed in motor neurons of patients carrying a polyglutamine expansion in the gene encoding ataxin-2 and cases displaying the G4C2-hexanucleotide repeat expansion in C9orf72 [348]. PARP-1 expression is also increased in astrocytes in sporadic ALS patients [349] and it is widespread in the cerebellum, motor cortex and parietal cortex, reflecting increased activation [348, 350]. PARP-1 levels are also elevated in astrocytes in the spinal cord in mutant SOD1^G93A^ transgenic mice [351]. Pharmacological inhibition of PARP inhibits the accumulation of stress-induced TDP-43 granules in the cytoplasm and toxicity in rat primary spinal cord cultures [348]. Furthermore, another study demonstrated that PARP1 knockout or treatment with PARP inhibitor olaparib reduces PAR levels and rescues TDP43-induced death in NSC-34 cells [352]. Inhibition of PARP-1 inhibits the ROS-induced cell death and suppresses mitochondrial ROS production via ATF4 and MAP kinase phosphatase-1 in human cell lines [353].

A significant increase in p53 expression has been detected in spinal cord tissues of ALS patients [354]. Similarly, increased nuclear p53 immunoreactivity was detected in the motor cortex and spinal ventral horn of post-mortem tissues from ALS patients [355] and in spinal motor neurons in SOD1^G86R^ mice [356]. Importantly, p53 knockout or knockdown extends the lifespan of a mouse model expressing poly(PR), and protects against neurodegeneration in *Drosophila* models [357]. Strikingly, p53 knockout also inhibits DNA damage in poly(PR)-transduced cells and C9orf72-ALS iPSC-derived motor neurons. Increased DNA damage was observed following the ectopic expression of poly(GR)_80_ or (GR)_80_ in iPSC-derived control neurons[13] and pharmacological or genetical reduction of oxidative stress partially retrieved DNA damage [13]. The adverse role of poly(GR) on DNA damage has also been confirmed in neuronal cells in *Drosophila* [358]. Together, these studies indicate that p53 mediates the C9orf72-DPR-induced toxicity upstream of DNA damage, rather than downstream, implying that redox homeostasis is crucial for regulation of p53 function, and its modulation may protect against DNA damage [357].

Expression of the C9orf72 repeat expansion induces DNA damage in familial ALS patient tissues and in cellular models [13, 347, 359]. Moreover, poly(GR)_80_ aggregates induce DNA damage and increase ROS levels in iPSC motor neurons derived from C9orf72-ALS/FTD patients, linking redox dysregulation to DNA damage [13]. Similarly, induction of oxidative stress and upregulation of DNA damage markers γH2AX, ATR, GADD45, and p53 were observed in an age-dependent manner in iPSC-derived C9orf72 motor neurons [13]. In the same study, cellular toxicity was rescued following administration of a water-soluble antioxidant and vitamin E analog, Trolox, in C9orf72 iPSCs. Furthermore, in another study, myogenic progenitors derived from C9orf72 ALS patients demonstrated high susceptibility to oxidative stress and dysregulation of mitochondrial and DNA repair genes, leading to cellular toxicity [360]. Similarly, modifiers of poly(GR)_100_ toxicity induce dysregulation of mitochondrial NADPH and DNA damage repair-related pathways in yeast cells [361]. Another study demonstrated increased mtDNA due to increased ROS specifically in C9orf72 ALS patient-derived fibroblasts, but not in TDP-43 A382T fibroblasts [362].

R-loops associated with oxidative stress are increased in post-mortem spinal cord tissues of C9orf72 patients and in poly(GA)-transfected MRC5 cells [359]. Both DNA damage and cell death in poly(GA)-expressing cells can be partly rescued by overexpressing senataxin, which resolves R-loops [359, 363]. Interestingly, mutations in the senataxin gene cause an autosomal dominant form of ALS, ALS4 [364]. Interestingly, there is evidence that sentataxin regulates redox homeostasis. An N-terminal truncation mutant of Sen1, the yeast homolog of human senataxin, is critical for cell survival through regulation of redox homeostasis. This mutant also displays severe loss of mtDNA [365]. Importantly, the N-terminal substrate interaction and C-terminal RNA/DNA helicase domains are conserved in Sen1, implying that the same domains may perform a similar function in human senataxin. Furthermore, sentataxin has 31 cysteine residues involved in disulphide bonding via redox-regulated PDI [366]. Moreover, residue C1554, which is expected to engage in disulphide linkage with C1509, is mutated in a sporadic case of ALS4 [367]. These findings together suggest that dysregulated redox signalling, leading to ROS production, is associated with C9orf72 in ALS.

While wild-type SOD1 is protective against DNA damage, ALS-mutant SOD1^G93A^ displays less nuclear localization and antioxidant activity and is not protective in cellular models [368]. SOD1^G93A^ expression in cells deficient in aprataxin, which facilitates SSB and NHEJ DNA repair, sensitises cells to oxidative stress, exacerbates DNA repair deficiencies and increases the levels of heterochromatin [369]. Another study demonstrated more DNA damage in peripheral blood mononuclear cells (PBMCs) of sporadic ALS patients, which display high levels of aggregated SOD1 compared to controls. However, no DNA damage was observed in PBMCs expressing soluble SOD1 only [370]. Increased levels of oxidative DNA damage, DNA strand breaks, p53 activity and apoptosis were detected in cells expressing mutant SOD1^G93A^ compared to wild-type SH-SY5Y cells [371]. However, in a more recent study, similar levels of DNA damage, assessed by the presence of γH2AX-positive foci, were detected in SOD1^G93A^ and SOD1^A4V^ patient-derived iPSC lines compared to isogenic controls [330].

TDP-43 is redox-regulated because the cellular redox conditions control its solubility and nuclear function [372]. We and others have demonstrated that TDP-43 normally functions in the repair of DSBs by NHEJ and associates with XRCC4 and ATP-dependent DNA Ligase 4 [329, 373]. However, this function is impaired by the ALS-associated mutations [332]. Moreover, GSH depletion by buthionine sulfoximine significantly increases mutant TDP-43^M337V^ mislocalisation and inclusion formation in Neuro-2a cells [12]. Redox dysfunction results in oxidation and phosphorylation of TDP-43 by GADD34, which is induced by DNA damage, leading to cytoplasmic mislocalisation in HEK293T cells [374]. Neuronal cells expressing mutant TDP-43 (Q331K and M337V) exhibit shortened neurites, increased oxidative stress and lower levels of heme oxygenase HO-1, which regulates redox signalling [375]. Hence, as TDP-43 is important in DNA repair, mutations in TDP-43 could lead to DNA damage and induce redox dysfunction.

FUS is recruited to oxidative DNA damage sites in response to DNA SSB formation, where it facilitates the recruitment of XRCC1 and nuclear ligase III to regulate its ligation activity for optimal BER activity [376]. Loss of nuclear FUS results in defects in DNA nick ligation in motor neurons due to reduced recruitment of XRCC1/ligase III to DNA strand breaks in cellular models [376]. Interestingly, PARP-dependent DNA damage and apoptosis have been detected in human iPSCs over-expressing mutant FUS-NLS, which induces FUS mislocalisation to the cytoplasm [377]. PARP is also involved in the formation of aberrant phase transition of FUS from the liquid compartments to solid-like aggregates, a process which is redox-regulated, at DNA damage sites [378–380]. In addition, FUS^R521C^ transgenic mice display both oxidative damage and defects in DNA ligation [381], implying that defects in DNA repair mechanisms and redox dysregulation are associated with FUS in ALS.

*APE1* is implicated as a possible ALS-associated gene that is upregulated in motor neurons of ALS patients [382–384]. Furthermore, the motor cortex of ALS patients contains epigenetic hypomethylation of the *APE1* promoter, and this region is vulnerable to DNA lesions induced by free radicals and intermediates [330]. In pre-symptomatic transgenic SOD1^G93A^ mice, expression of APE1 is reduced in spinal motor neurons, indicating that a deficiency in DNA repair precedes motor neuron degeneration [385]. However, whether the APE1 redox and DNA repair activity are dysregulated in ALS is unknown.

### HD

HD is a severe, rapidly progressing autosomal-dominant condition caused by expansion of CAG (encoding glutamine) repeats in the gene encoding huntingtin protein [386]. Translation of the polyglutamine repeat then produces an abnormally long protein. HD involves motor, psychiatric and cognitive symptoms and it results from degeneration of neurons in the striatum and other regions of the cerebral cortex. Unaffected individuals possess less than 35 polyglutamine repeats, whereas HD patients normally possess 36 to 120 repeats. Interestingly, the number of repeats correlates inversely with the age of disease onset, implying that disease is dependent on repeat length [387]. Similar to other neurodegenerative disorders, redox dysregulation and impaired DNA repair are implicated in the pathogenesis of HD.

DNA damage is strongly implicated in the etiology of HD [388]. Elevated DNA damage has been detected in human HD fibroblasts and HD mouse models [389], where it precedes the aggregation of mutant huntingtin [389]. More DNA damage has also been detected in HD patient PBMCs compared to controls [390] and another clinical study also reported increased DNA damage in prodromal HD in blood cells [391]. ATM was also identified as a modifier of HD-relevant phenotypes in a mouse model [392]. More recently, many genes involved in DNA repair were found to be important regulators of age of disease onset and severity in a large genome-wide association study, including FANCD2/FANCI-associated nuclease 1(*FAN1*) and *ERCC3* (ERCC excision repair 3)*.* Also, defects in DNA repair pathways, including inactivation of DNA mismatch repair genes such as MutS Homolog 3 (*MSH3*), were associated with modification of age of onset in multiple CAG repeat expansion diseases, suggesting that the CAG repeat itself is the cause of modification [393]. The most significant hit was FAN1, which is associated with ICL repair, and multiple MMR genes were also detected [388]. Moreover, several of the genes identified are also related to mitochondrial and redox signalling pathways [388].

There is also evidence for oxidative DNA damage in HD, as revealed by increased expression of 8-OHdG compared to controls, in both nuclear DNA and mtDNA [394], further linking DNA damage to redox dysregulation. HD patient fibroblasts also display deficient repair following oxidative DNA damage [389]. Furthermore, somatic expansion of the polyglutamine repeat has also been associated with BER, which is a naturally error-prone process. This involves the BER enzyme OGG1 and the removal of oxidized base lesions, resulting in somatic expansion of repeats by SSBs and strand slippage [386]. In addition, the extent of oxidative damage correlates positively with the expansion length [386, 387]. Furthermore, the somatic expansion process induces further oxidative damage and error-prone repair of this damage by the formation of longer repeats [386], forming a vicious, escalating oxidation–BER cycle. These data therefore imply that accumulation of oxidative DNA lesions due to dysfunction of mutant huntingtin in DNA repair in conditions of ROS may contribute to the onset of HD [389].

The normal huntingtin protein is also thought to function in DNA repair, where it is detected as part of the transcription-coupled repair (TCR) complex. TCR is a subtype of NER that rapidly removes specific types of DNA damage from transcribed strands of expressed genes, in contrast to non-transcribed strands. The TCR complex detects lesions and mediates repair during transcription. Mutant huntingtin impairs the function of components of the TCR complex, PNKP and ataxin-3, resulting in more DNA damage and ATM hyperactivation [395].

Huntingtin protein is also thought to repair damaged DNA following oxidative stress, revealing that redox dysregulation and DNA repair are intimately linked in HD [389]. Huntingtin localises and forms a scaffold at DNA damage sites via an ATM-dependent process in the presence of ROS. Huntingtin protein is a sensor of ROS and it relocates to the nucleus following oxidation of Met8 [396]. In the presence of ROS, liquid–liquid phase-separated droplets containing huntingtin colocalised with ATM are increased [396–398]. ATM is activated during oxidative stress [399] and inhibiting its activity delays disease progression in mouse HD models [392].

Several studies have reported oxidative damage, decreased levels of antioxidants, cysteine and vitamin C, and deposition of iron (Fe) in the cytoplasm and mitochondria, in cells and tissues from HD models and patients. Uptake of vitamin C is compromised in cellular and mouse models of HD, which precedes mitochondrial dysfunction [400]. The levels of GSH are dysregulated in the plasma and cortex of HD patients compared to controls [401, 402], although whether there is an increase or decrease in GSH is still under debate [403]. Nevertheless, both processes would perturb the cellular redox conditions in HD. There is also evidence for impaired cysteine metabolism in HD [16].

Whilst the HD repeat length is the main factor determining the age at disease onset, genetic modifiers also make a significant contribution to the variation in onset age [404]. The expanded CAG polyglutamine repeat is somatically unstable, and its length increases progressively over time in neurons, particularly in the striatum and cortex. Due to this somatic instability, larger increases in repeat length are associated with earlier disease onset [404]. Interestingly, somatic expansion of the HD CAG repeat is mediated by DNA damage, via a mechanism involving the introduction of mutations by MMR [388]. Similarly, in cellular and animal models, deficiency of MSH3, MSH2, MLH3, MLH1 or PMS2, or increased expression of FAN1, prevents the somatic expansion of CAG repeats [405, 406]. Similarly, transcriptome-wide association studies have revealed that lower expression of MSH3, and increased levels of FAN1, are associated with CAG repeat stability, later onset, and slower disease progression [407, 408]. However, it is unclear if this involves oxidative DNA damage. A more recent study showed that an interaction between FAN1 and MLH1, via a highly conserved SPYF motif at the N terminus of FAN1, is protective against somatic expansion by restricting the recruitment of MLHA by MSH3 [405]. This study suggested that FAN1 normally stabilizes CAG repeats, by both inhibiting formation of the MMR complex that promotes somatic repeat expansion and enhancing correct DNA repair via nuclease activity.

## The impact of aging on redox regulation and DNA repair processes

The biggest risk factor for neurodegenerative diseases is aging [409, 410]. Importantly, age-related decline is also a critical socio-economic challenge world-wide due to the increasing aging population [411]. Thus, the incidence of neurodegenerative conditions is likely to increase significantly in the coming decades. Aging is a complex event characterised by damage to both proteins and DNA and during this process cells become senescent, whereby they lose the ability to grow and divide. Aging theories are associated with a decline in cellular function and the accumulation of damage, involving either programmed aging or failure accumulation. The latter theory states that aging is a consequence of the accumulation of damage to cellular components. Interestingly, both oxidative stress and DNA damage increase significantly during aging due to decreases in the efficiency of DNA repair and in the maintenance of redox homeostasis [409, 412]. Moreover, oxidative damage to DNA increases during aging, thus together these mechanisms are central contributors to the normal aging process. Similarly, during aging, the efficiency of proteostasis declines, leading to its ‘collapse’ [409, 413, 414]. While historically maintenance of the genome and the proteome were considered separate processes, emerging evidence reveals that they are much more inter-related than previously recognized [415, 416]. Hence, it is probable that redox dysregulation, DNA damage, and proteostasis decline together form a viscous cycle, driving or exacerbating the aging process [415, 416]. In addition, environmental factors such as UV and other forms of radiation, toxic chemicals, and heavy metals, are also strongly implicated in aging.

During normal aging, increased cellular GSSG/GSH ratio and more GSH oxidation are present, leading to increased levels of 8-OHdG [417]. The formation of 8-oxoG lesions increases with aging [248, 250, 418]. A recent study proposed a mechanism which links 8-OHdG elevation to aging [419]. This study demonstrated that histone deacetylase 1 activation results in attenuated accumulation of oxidative lesions in both aged mice and 5×FAD mice [419]. Mice with knockout of *slc25a46* (solute carrier family 25 member 46), a nuclear gene encoding mitochondrial transmembrane protein, display premature aging phenotypes characterized by shortened life span, defective motor ability and redox imbalance in the brain, and neuropathy [420]. This finding implies that acceleration of redox dysregulation with aging may indirectly lead to increased neuronal loss and neurodegeneration. An interesting link between aging, neurodegeneration and NER has also been described, with a possible relationship to ALS. A mouse model with a hypomorphic mutation in *Ercc1*, *Ercc1*Δ/-, displays degeneration of motor neurons and shortened lifespan [421]. Together, these studies imply that aging, redox dysregulation, and DNA damage are closely associated and compound during neurodegeneration.

## Genetic modifications to DNA repair proteins—what do they reveal about redox regulation, DNA damage and neurodegeneration?

This extensive evidence linking DDR defects to neurodegenerative diseases implies that neurons are highly susceptible to DNA damage, particularly oxidative DNA lesions. Furthermore, DSBs are now known to form and modify gene expression during physiological neuronal activity [422, 423], which may mediate neuronal plasticity [423]. However, it is also important to consider the relationship between genetic defects in DDR proteins and how this is related to neurodegeneration.

DNA repair syndromes are familial conditions characterized by mutations in DDR proteins. They include Cockayne syndrome, ataxia telangiectasia (AT), xeroderma, pigmentosum, trichothiodystrophy, and Nijmegen breakage syndrome [424]. Whilst heterogeneous, these disorders commonly display neurological features, particularly microcephaly and ataxia [425], as well as phenotypes associated with accelerated aging, the biggest risk factor for neurodegeneration. Furthermore, some of these syndromes also directly involve neuronal degeneration, such as AT, which displays progressive degeneration of both Purkinje and granule neurons in the cerebellum. Moreover, in some instances, the defect is almost exclusively neurological, such as ataxia with oculomotor apraxia type 1 (AOA1), AOA5 [426, 427], and spinocerebellar ataxia with axonal neuropathy 1 (SCAN1) [425]. These findings thus raise the possibility that DNA damage arising in neurons can induce human diseases.

Further evidence for this hypothesis comes from the detection of single nucleotide polymorphisms (SNPs) in neurodegenerative disorders. SNPs involve substitution of a single nucleotide in DNA, and they arise frequently within the genome [428]. They have been detected in multiple DDR genes in neurodegenerative diseases, including those that repair oxidative DNA damage. In AD, SNPs in BER genes encoding APE1, OGG1, NEIL1, flap endonuclease 1, or DNA ligases I or III, have been associated with increased risk of disease [429, 430], although this was disputed in another study [431]. Similarly, in PD, *APE1* and *OGG1* polymorphisms have been observed [432, 433]. Furthermore, in ALS, a Ser326Cys polymorphism in *OGG1* is associated with an increased risk of sporadic ALS [342], and several variants in *APE1* have previously been described [383]. SNP modifiers in the *HTT* gene impair NF-κB binding and regulate DNA activity in HD patients [434]. However, investigations in larger populations and more extensive genetic studies are required to confirm the links between SNPs and neurodegenerative diseases. It also remains debatable whether these polymorphisms are independent disease risk factors.

While these observations strongly link DNA damage to human neurological diseases, it has been difficult to establish whether defects in the DDR directly cause neurodegeneration. Nevertheless, recent observations that proteins centrally involved in neurodegeneration also function in DNA repair—particularly TDP-43, FUS, and huntingtin—imply that genetic defects in the DDR directly induce neuronal degeneration and/or death. It is noteworthy that TDP-43, FUS, and huntingtin all function in the repair of oxidative DNA damage, implying that impairment in redox-dependent DNA repair mechanisms is capable of inducing human neurodegenerative diseases. In addition, a recent study provided further evidence for this hypothesis. Enhancing the repair of oxidative DNA damage by overexpressing APE1 and OGG1 was protective against neuronal apoptosis in mouse models [435]. Hence, this finding implies that the accumulation of oxidative DNA damage directly induces neuronal degeneration and death.

However, it should be noted that there are also significant differences between DNA repair syndromes and neurodegenerative disorders. DNA repair syndromes typically first appear in early childhood, whereas most neurodegenerative conditions present in mid-late adulthood. Indeed, microcephaly, which is present in a significant proportion of DNA repair syndromes, results from abnormal embryonic development. These differences, however, may reflect the nature of the specific mutation, the DDR pathway involved, and/or its degree of impact on DNA repair in neurons. Some genetic DDR defects may hasten the development of age-associated phenotypes to such an extent that DNA repair syndromes arise in childhood rather than in adulthood. In contrast, other defects may be less severe, and require the contribution of additional genetic or environmental factors before disease manifests, such as in neurodegenerative disorders. Consistent with this notion, recent studies have shown that neurodegeneration is a multistage process, whereby several sequential steps are required before disease manifests: six for ALS [436] and PD [437], and 14 for AD [438]. Thus, SNPs or genetic defects combined with environmental factors, oxidative stress and aging, may together be required for the development and progression of neurodegenerative diseases [439, 440]. Furthermore, it is possible that defects in DNA repair can both directly cause neurodegeneration and/or contribute to neuronal death in combination with other factors.

Interestingly, DNA repair syndromes displaying mutations in SSBR genes commonly result in neurodegeneration. AOA1, AO4, SCAN1, and AO5 display variations in genes encoding proteins that function in BER or SSB repair (*APTX*, *PNKP*, *TDP1* and *XRCC1*, respectively)[435]. These findings may reflect the post-mitotic nature of neurons. SSBs arising in cells actively undergoing the cell cycle often convert to DSBs, which can be subsequently repaired by error-free HR, unlike in neurons. Hence, it is possible that neurons are particularly sensitive to mutations affecting SSBR. Furthermore, mutations to some DNA repair genes are embryonically lethal in mice, particularly those encoding BER proteins. This includes APE1, thymine DNA glycosylase, and DNA ligase IV [441, 442]. This implies that oxidative DNA damage cannot be tolerated in neurons, thus the most severe mutations do not manifest in humans because they are eliminated from the gene pool. This further highlights the important relationship between oxidative DNA damage and neuronal viability.

Nevertheless, it remains unclear why mutations within proteins involved in the same DNA repair pathways can lead to widely different pathologies. It is possible that the selective vulnerabilities of distinct neuronal subtypes to different repair deficits play a role. An interesting observation is that neurodegeneration in AT, AOA1, and SCAN1 is restricted primarily to neurons localised in the cerebellum, whereas in AD, PD, ALS and HD, a more diverse range of neuronal subtypes are targeted. However, impairment in motor control and posture are present in neurodegenerative diseases and the role of the cerebellum in the regulation of motor control is well established. Moreover, cerebellar dysfunction is now implicated in PD, HD, AD [443] and ALS [444], implying that neurodegenerative disorders are not as distinct from DNA repair syndromes as they may initially appear.

## Conclusion

The pathways linking redox dysfunction and DNA damage are complex and we are only just beginning to unravel their full complexity. The most well characterised of these mechanisms involve DNA damage induced by oxidative stress. Furthermore, it has also been recognised for some time that several proteins, primarily APE1 and p53, possess both redox activity and DNA repair functions. However, an increasing number of DNA repair proteins are now known to also possess redox activity. Similarly, a growing list of redox regulatory proteins is now known to function in the DDR. It is possible that some of these proteins act indirectly, by modulating redox conditions in the nucleus, or by modulating expression of DNA repair proteins. Likewise, several transcription factors that function in the DDR are known to be regulated by redox processes. However, it is also possible that these proteins may function directly in DNA repair, although these mechanisms remain poorly understood. Nevertheless, it is becoming apparent that there are much more extensive links between the DDR and redox signalling than previously recognised.

**Fig. 5:**
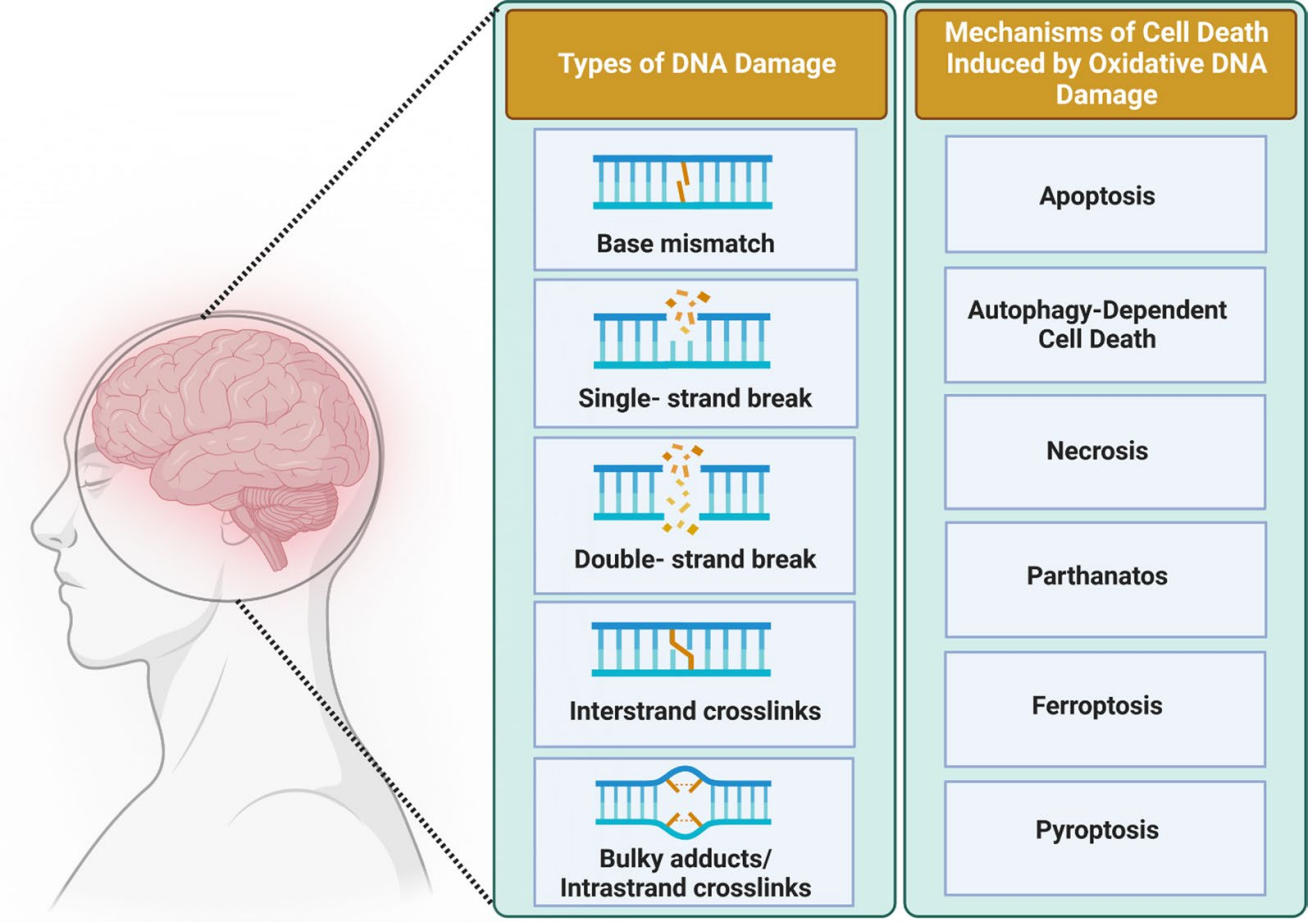
Types of DNA damage induced by oxidative stress in neurodegenerative diseases. DNA damage can induce diverse cell death mechanisms such as apoptosis, autophagy-dependent cell death, necrosis, parthanatos, ferroptosis, pyroptosis and lysosome-dependent cell death. However, not all these mechanisms are associated with neurodegenerative diseases

Neurons are terminally differentiated and thus cannot dilute the effects of DNA lesions by cell division like other cell types. Defective DNA repair therefore has potentially catastrophic effects on neurons and is increasingly implicated in neurodegeneration. Neurons are very metabolically active and thus generate high levels of ROS, and similarly redox dysregulation is also implicated in the pathogenesis of these disorders. Thus, DNA repair imposes additional energetic stress onto neurons given their high rates of metabolism. As energy is depleted, ROS by-products cause further damage. Both DNA damage and redox dysregulation become impaired during the aging process, which may promote neurodegeneration. In addition, impaired levels of GSH have been reported in various neurodegenerative disorders including AD, PD, HD, and ALS. However, while environmental factors are known to induce DNA damage and oxidative stress, the evidence for their role in many neurodegenerative conditions is conflicting [445]. Mitochondrial function also becomes impaired during normal human aging, and damage to both nuclear DNA and mtDNA is implicated in neurodegeneration. Redox regulation controls epigenetic mechanisms, which may also contribute to neurodegeneration. Together, these factors are likely to combine and provide an ever-increasing threat to neurons, which struggle to maintain their integrity over time. Eventually, even minor impairments in DNA repair or redox dysregulation can have serious consequences and lead to neurodegeneration (Fig. 5).

Understanding the poorly characterised connections between oxidative stress and DNA damage is necessary for a better understanding of disease mechanisms in neurodegenerative diseases, given this is a primary source of DNA lesions in neurons. Moreover, there are no known mechanisms to repair oxidative (or other) DNA damage in neurodegenerative disorders. Hence, improving our knowledge of these relationships may ultimately lead to the design of better therapeutic strategies, based on preventing both redox dysregulation and DNA damage.

## Data Availability

Not applicable.

## Abbreviations

ROS: Reactive oxygen species
RNS: Reactive nitrogen species
DDR: DNA damage response
AD: Alzheimer’s disease
PD: Parkinson’s disease
ALS: Amyotrophic lateral sclerosis
HD: Huntington’s disease
RSS: Reactive sulphur species
FTD: Frontotemporal dementia
SOD: Superoxide dismutases
GPx: Glutathione peroxidase
GR: Glutathione reductase
Prxs: Peroxiredoxins
Nrf2: Nuclear factor erythroid 2-related factor 2
NOS: Nitric oxide synthase
GSH: Glutathione
TrxR: Thioredoxin reductase
APE1: Apurinic/apyrimidinic endonuclease 1
TXNIP: Thioredoxin-interacting protein
AIF: Apoptosis inducing factor
PDI: Protein disulphide isomerase
UPR: Unfolded protein response
OXPHOS: Oxidative phosphorylation
mtDNA: Mitochondrial DNA
XOR: Xanthine oxidoreductase
DSB: Double-stranded break
ICL: Inter-strand crosslink
PAH: Polycyclic aromatic hydrocarbon
BER: Excision repair
NER: Nucleotide excision repair
PNKP: Polynucleotide kinase phosphatase
TDP1: Tyrosyl DNA phosphodiesterase 1
8-oxoG: 8-Oxoguanine
TOP: Topoisomerase
MMC: Mitomycin C
PUFA: Polyunsaturated fatty acid
MDA: Malondialdehyde
PI3K: Phosphatidylinositol 3-kinase
ATM: Ataxia telangiectasia mutated kinase
ATR: Rad3-related protein
OGG1: 8-OxoG glycosylase
PCNA: Proliferating cell nuclear antigen
GG-NER: Global genome NER
TC-NER: Transcription-coupled NER
RPA: Replication protein A
ERCC1: Excision repair protein-1
FA: Fanconi anemia
SSBR: Single-strand break repair
PARPs: Poly-ADP-ribose-polymerases
PNK: Polynucleotide kinase
HDR: Homology directed repair
NHEJ: Non-homologous end joining
H2AX: H2A histone family member X
CtIP: CtBP-interacting protein
MGMT: O-6-methylguanine-DNA methyltransferase
MCI: Mild cognitive impairment
8-OHA: 8-Hydroxyadenine
8-OHdG: 8-Hydroxy-2’-deoxyguanosine
SNpc: Substantia nigra pars compacts
UA: Uric acid
6-OHDA: 6-Hydroxydopamine
C9orf72: Chromosome 9 open reading frame 72
TDP-43: TAR DNA-binding protein-43
FUS: Fused in sarcoma
PBMC: Peripheral blood mononuclear cell
XRCC4: X-ray repair cross-complementing protein 4
FAN1: FANCD2/FANCI-associated nuclease 1
ERCC3: ERCC excision repair 3
MSH3: MutS Homolog 3
TCR: Transcription-coupled repair
AT: Ataxia telangiectasia
AOA1: Ataxia oculomotor apraxia type 1
SCAN1: Spinocerebellar ataxia with axonal neuropathy 1
